# Isoflurane activates the type 1 ryanodine receptor to induce anesthesia in mice

**DOI:** 10.1371/journal.pbio.3003172

**Published:** 2025-06-03

**Authors:** Hiroyuki J. Kanaya, Ken Kuwajima, Yuko Ito, Yuta Shinohara, Yohei Okubo, Shinnosuke Shiono, Fumiya Tatsuki, Rei-ichiro Ohno, Hideki Ukai, Maki Ukai-Tadenuma, Kenta Sumiyama, Hiroshi Fujishima, Rikuhiro G. Yamada, Daisuke Tone, Hiroshi Kiyonari, Masaki Kikuchi, Takashi Umehara, Takashi Murayama, Kazunori Kanemaru, Masamitsu Iino, Koji L. Ode, Takatsugu Hirokawa, Hiroki R. Ueda

**Affiliations:** 1 Department of Systems Pharmacology, Graduate School of Medicine, The University of Tokyo, Tokyo, Japan; 2 Cellular and Molecular Biotechnology Research Institute, National Institute of Advanced Industrial Science and Technology, Tokyo, Japan; 3 Laboratory for Synthetic Biology, RIKEN Center for Biosystems Dynamics Research, Suita Osaka, Japan; 4 Department of Cellular and Molecular Pharmacology, Juntendo University Graduate School of Medicine, Tokyo, Japan; 5 Laboratory for Mouse Genetic Engineering, RIKEN Center for Biosystems Dynamics Research, Suita, Osaka, Japan; 6 Department of Systems Biology, Institute of Life Science, Kurume University, Kurume, Fukuoka, Japan; 7 Laboratory for Animal Resources and Genetic Engineering, RIKEN Center for Biosystems Dynamics Research, Chuo-ku, Kobe, Japan; 8 Laboratory for Epigenetics Drug Discovery, RIKEN Center for Biosystems Dynamics Research, Tsurumi, Yokohama, Japan; 9 Department of Physiology, Nihon University School of Medicine, Itabashi-ku, Tokyo, Japan; 10 Division of Biomedical Science, Institute of Medicine, University of Tsukuba, Tsukuba, Ibaraki, Japan; 11 Transborder Medical Research Center, University of Tsukuba, Tsukuba, Ibaraki, Japan; Columbia University Irving Medical Center, UNITED STATES OF AMERICA

## Abstract

Inhaled anesthetics were first introduced into clinical use in the 1840s. Molecular and transgenic animal studies indicate that inhaled anesthetics act through several ion channels, including γ-aminobutyric acid type A receptors (GABA_A_Rs) and two-pore domain K^+^ (K2P) channels, but other targets may mediate anesthetic effects. Mutations in the type 1 ryanodine receptor (RyR1), which is a calcium release channel on the endoplasmic reticulum membrane, are relevant to malignant hyperthermia, a condition that can be induced by inhaled anesthetics. However, it was previously uncertain whether inhaled anesthetics directly interact with RyR1. In our study, we demonstrated that isoflurane and other inhaled anesthetics activate wild-type RyR1. By employing systematic mutagenesis, we discovered that altering just one amino acid residue negates the response to isoflurane, thus helping us to pinpoint the potential binding site. Knock-in mice engineered to express a mutant form of RyR1 that is insensitive to isoflurane exhibited resistance to the loss of righting reflex (LORR) when exposed to isoflurane anesthesia. This observation suggests a connection between RyR1 activation and the anesthetic effects in vivo. Moreover, it was shown that RyR1 is involved in the neuronal response to isoflurane. Additionally, administering new RyR1 agonists, which share the same binding site as isoflurane, resulted in a sedation-like state in mice. We propose that isoflurane directly activates RyR1, and this activation is pertinent to its anesthetic/sedative effects.

## Abstract 2

What are the molecular targets of inhaled anesthetics, such as isoflurane? This study reports that isoflurane binds type 1 ryanodine receptor, and determines that genetically disrupting a specific residue or pharmacologically activating this binding site with novel agonists can modulate sensitivity to anesthesia.

## Introduction

Inhaled anesthetics are volatile compounds that produce general anesthesia. Their use as general anesthetics began in the 1840s when their anesthetic properties were demonstrated [1–3]. For decades, researchers have been investigating the molecular targets of these anesthetics [4–6]. Many proteins are sensitive to inhaled anesthetics, while a modest number of ion channels mediate their neurobehavioral effects. For instance, γ-aminobutyric acid type A receptors (GABA_A_Rs) are direct targets of inhaled anesthetics and are significant concerning their effects in vivo [7–10]. Furthermore, inhaled anesthetics activate two-pore K^+^ (K2P) channels, a group of K^+^ leak channels [11–13]. Multiple lines of evidence suggest that the activation of K2P channels plays a role in mediating the effects of inhaled anesthetics [14–19]. Inhaled anesthetics also have additional substrates, such as certain cation channels, which may be relevant to their anesthetic properties [20]. Previous studies have also suggested presynaptic mechanisms of inhaled anesthetics. It was reported that isoflurane, an inhaled anesthetic inhibits synaptic vesicle exocytosis by inhibiting presynaptic calcium influx [21]. Given the complexity of the molecular mechanisms of inhaled anesthetics, their anesthetic effects may involve molecular targets that have not yet been identified.

**Fig 1:**
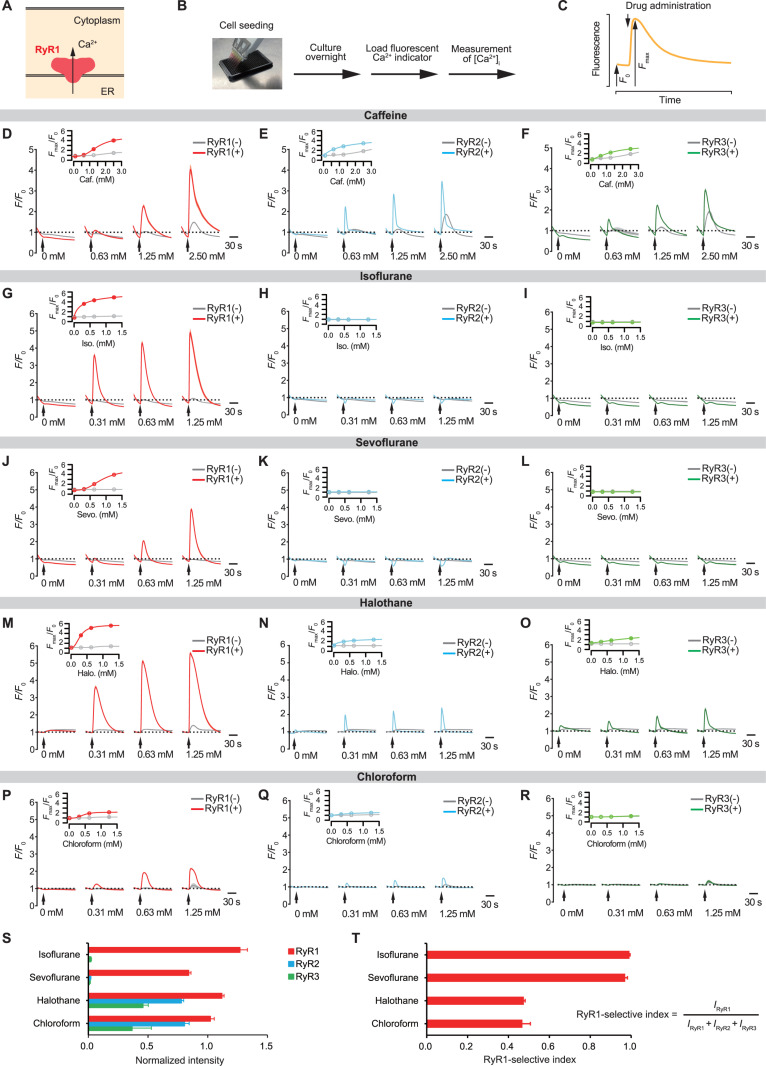
Inhaled anesthetics activate RyR1. **(A)** RyR1 is a calcium release channel located on the membrane of the endoplasmic reticulum (ER). **(B)** Experimental method for monitoring intracellular calcium ([Ca^2+^]i). **(C)** Quantification of the intracellular calcium signal. *F*_0_ represents the averaged intensity of baseline fluorescence, whereas *F*_max_ represents the peak intensity following the drug administration. **(D–F)** Caffeine responses for RyR1, RyR2, and RyR3. The time-course reaction (*F/F*_0_) and the peak (*F*_max_/*F*_0_) are plotted for conditions with and without expression. Caffeine was administered after measuring the baseline fluorescence for 30 s, as shown by the arrows. *N* = 4. **(G–I)** Response of RyR1, RyR2, and RyR3 to isoflurane. *N* = 4. **(J–L)** Response of RyR1, RyR2, and RyR3 to sevoflurane. *N* = 4. **(M–O)** Response of RyR1, RyR2, and RyR3 to halothane. *N* = 4. **(P–R)** Response of RyR1, RyR2, and RyR3 to chloroform. *N* = 6. **(S)** Normalized peak intensity for each anesthetic (1.25 mM). Data are normalized so that the peaks of 2.50 mM caffeine and the control (without pharmacological agents) are 1.0 and 0, respectively, for each isoform (equation 1 in Materials and methods). *N* = 4–6. **(T)** RyR1-selective index. *I*_x_ represents the normalized peak intensity at 1.25 mM anesthetics for each isoform. *N* = 4–6. Data are represented as Mean ± SD. For the insets of panels **D**–**R**, the data were fitted with logistic functions (equation 4 in Materials and methods). RyR1, the type 1 ryanodine receptor; ER, endoplasmic reticulum; RyR2, the type 2 ryanodine receptor; RyR3, the type 3 ryanodine receptor; Caf., Caffeine; Iso., Isoflurane; Sevo., Sevoflurane; Halo., Halothane.

The type 1 ryanodine receptor (RyR1) is a promising candidate as a target for inhaled anesthetics because mutations of RyR1 are linked to malignant hyperthermia (MH), a potentially life-threatening pharmacological disorder induced by the use of inhaled anesthetics [22,23]. RyR1 is an intracellular homotetrameric calcium release channel located on the membrane of the endoplasmic reticulum (ER) and the muscle equivalent, the sarcoplasmic reticulum (SR), along with the other isoforms (RyR2 and RyR3) (Fig 1A). RyRs release calcium into the cytosol from intracellular calcium stores in response to cytosolic calcium, which is known as calcium-induced calcium release (CICR). In many instances, the RyR1 mutants associated with malignant hyperthermia susceptibility (MHS) are linked to the abnormal increase in CICR activity [24]. However, it was unclear whether inhaled anesthetics interact directly with RyR1.

The connection between RyR1 and inhaled anesthetics might extend beyond the cause of MH. A recent study reported that when treated with an inhaled anesthetic, MHS mice transition to a flat electroencephalogram (EEG) more quickly than non-MHS mice before showing severe MH symptoms [25]. This suggests that they enter a state of deep anesthesia more rapidly. In addition, the sole ortholog of *Ryr* in *Drosophila* mediates behavioral responses to inhaled anesthetics [26]. These findings suggest that RyR1 may be significant not only to MHS but also to the anesthetic actions of inhaled anesthetics.

In this study, through a systematic analysis of each mammalian RyR isoform, we showed that isoflurane, an inhaled anesthetic, activates wild-type RyR1 at clinically relevant concentrations. We identified M4000 as a key residue for the response to isoflurane and determined the putative binding site. Knock-in (KI) mice expressing the mutant RyR1, which is insensitive to isoflurane, were resistant to isoflurane anesthesia, as evidenced by a shift in the neurophysiological signature of deep anesthesia. Neuronal RyR1 inhibition altered the response to isoflurane in vitro and in vivo. Furthermore, we identified novel RyR1 agonists that share the binding site with isoflurane by large-scale in silico chemical screening. The novel agonists were effective in inducing a sedation-like state in mice and sensitizing the anesthetic action of isoflurane. Thus, RyR1 is a functional target of isoflurane that is directly related to its anesthetic properties.

## Results

### Isoflurane activates RyR1

There are three isoforms of RyRs in mammals (RyR1, RyR2, and RyR3). To systematically examine the pharmacological properties of each isoform, we used HEK 293 cell lines that express rabbit isoforms under tetracycline regulation [27]. These cells were seeded in a 384-well plate and subjected to an assay in which the intracellular calcium levels were measured using a fluorescent calcium indicator upon drug administration (Fig 1B and 1C). While caffeine is a stimulant that antagonizes the adenosine receptors (IC_50_s on adenosine receptors are ~10 μM) [28,29], it is also a typical agonist of RyRs activating all isoforms at higher dosages [30,31]. Caffeine administration caused dose-dependent increases in intracellular calcium levels depending on the tetracycline-mediated expression of each isoform (Figs 1D–1F and S1A). We observed that, while caffeine causes calcium elevation in the nonexpressed controls, most likely due to the presence of endogenous RyRs in HEK 293 cells [32], the distinct signals in the expressed conditions indicate that each isoform is successfully expressed as functional channels.

We then gave three different doses of inhaled anesthetics (0.31, 0.63, and 1.25 mM), as well as vehicle control (0 mM). Although the clinically effective dosages differ between inhaled anesthetics, the administered dosages cover or approach the aqueous concentrations corresponding to their minimum alveolar concentrations (MACs). We found that the administration of isoflurane, a frequently used inhaled anesthetic, strikingly elevated the intracellular calcium level at submillimolar concentrations depending on the expression of RyR1 (Fig 1G). The EC_50_ for isoflurane action on RyR1 was 203 μM (95% confidence interval [CI] = 182–224) (S1B Fig), which is a clinically relevant concentration (approximately 0.7 MAC). RyR2- and RyR3-expressing cells showed no detectable calcium increases in response to isoflurane (Figs 1H, 1I, and S1B). Similarly, sevoflurane, an inhaled anesthetic with clinical use, activated RyR1 but not RyR2 and RyR3 (Fig 1J–1L). We noted that, while the MAC of sevoflurane is 0.33 mM in aqueous solution, the similar dosage had no significant effect on RyR1 (Fig 1J). Halothane and chloroform, formerly used inhaled anesthetics, also activated RyR1 with minor effects on RyR2 and RyR3 (Fig 1M–1R), although chloroform’s actions were relatively small for all isoforms. The EC_50_s for halothane and chloroform were 288 μM (95% CI = 278–298) and 430 μM (95% CI = 414–447), corresponding to 1.3 MAC and 0.4 MAC, respectively. We normalized the maximum fluorescence intensity elicited by each anesthetic using the amount of the caffeine response (i.e., functionally normalizing fluorescent signals by the expression level of each isoform) (Fig 1S and equation 1 in Materials and methods) and quantified RyR1 selectivity (Fig 1T). Interestingly, while modern anesthetics isoflurane and sevoflurane (halogenated ethers) were highly selective for RyR1, the older anesthetic halothane (halogenated alkane) and the archaic anesthetic chloroform were less selective for RyR1.

To confirm that the calcium elevation induced by inhaled anesthetics comes from intracellular calcium stores, we performed the experiments under a nominal calcium-free environment. In the absence of extracellular calcium ([Ca^2+^]_E_ = 0 mM), caffeine, and isoflurane-induced calcium elevations were comparable to or even greater than those observed in the control condition ([Ca^2+^]_E_ = 1.26 mM) (S1C and S1D Fig). This suggests that the calcium increases caused by caffeine and isoflurane primarily derive from intracellular calcium stores rather than the extracellular space. To deplete the calcium storage in the ER, thapsigargin (TG), an inhibitor of the Sarco/ER Ca^2+^ ATPase, was given under the nominal calcium-free condition (S1E and S1F Fig). Ionomycin is a calcium ionophore that induces calcium release from intracellular stores to the cytosol as well as calcium influx from the extracellular space. Ionomycin-induced calcium elevation was substantially reduced in the TG-treated condition, regardless of RyR1 expression, confirming the effective depletion of ER calcium storage. TG also eliminated RyR1-dependent responses to caffeine and isoflurane. This indicates that calcium elevations observed during caffeine and isoflurane stimulation come from the TG-sensitive ER calcium store. The restoration of extracellular calcium (administration of 1.26 mM Ca^2+^) increased the intracellular calcium level via the mechanism of store-operated calcium entry, ensuring cell survival following TG treatment.

Next, we analyzed whether RyR1 inhibitors prevented isoflurane-induced calcium elevation. Treatment with dantrolene, a RyR inhibitor, significantly reduced the caffeine action on RyR1 and RyR3, but not on RyR2 (S2A–S2D Fig), consistent with the previously reported isoform selectivity [33]. Dantrolene also inhibited isoflurane-induced calcium elevation in RyR1-expressing cells (S2B Fig), indicating that the calcium signal is mediated by RyR1. Furthermore, we expressed a brain-specific splicing variant of RyR1 (RyR1-BsSV) in cells that express each RyR isoform (S2E Fig). This variant acts as a dominant negative form of RyR1 [34,35]. Compared to control cells expressing either mock or mCherry, RyR1-BsSV reduced the response of RyR1 to isoflurane, but it did not significantly impact the response to caffeine (S2F Fig). We observed that RyR1-BsSV expression did not consistently inhibit the caffeine responses of RyR2 and RyR3 compared to the controls expressing mock and mCherry (S2G and S2H Fig). These findings affirm the RyR1’s role in the calcium release induced by isoflurane.

Along with RyRs, inositol 1,4,5-trisphosphate receptors (IP_3_Rs) are calcium-releasing machinery on the ER membrane. To investigate the possible role of IP_3_Rs in the isoflurane-induced calcium release, we attempted to inhibit IP_3_Rs. IP_3_ 5-phosphatase (5ppase) hydrolyzes IP_3_, thereby inhibiting IP_3_Rs, however, an amino acid substitution mutant 5ppase(R343A/R350A) lacks the enzymatic activity [36,37]. Adenosine triphosphate (ATP) is recognized for stimulating IP_3_Rs by activating P2Y receptors on the cell membrane, which leads to the production of IP_3_ [38]. In contrast to the expression of 5ppase(R343A/R350A), expressing 5ppase(WT) led to a diminished response to ATP (S2I–S2K Fig), indicating successful inhibition of IP_3_Rs. Although the response to caffeine was slightly decreased under high dosage conditions (S2L Fig), there was no significant effect on the response to isoflurane (S2M Fig). This indicates that inhibiting IP_3_Rs does not change the response to isoflurane, suggesting that IP_3_Rs are not involved in the calcium release triggered by isoflurane. Overall, isoflurane causes calcium release from ER calcium stores by selectively activating RyR1.

### Identification of the RyR1 residues responsible for the response to isoflurane

**Fig 2:**
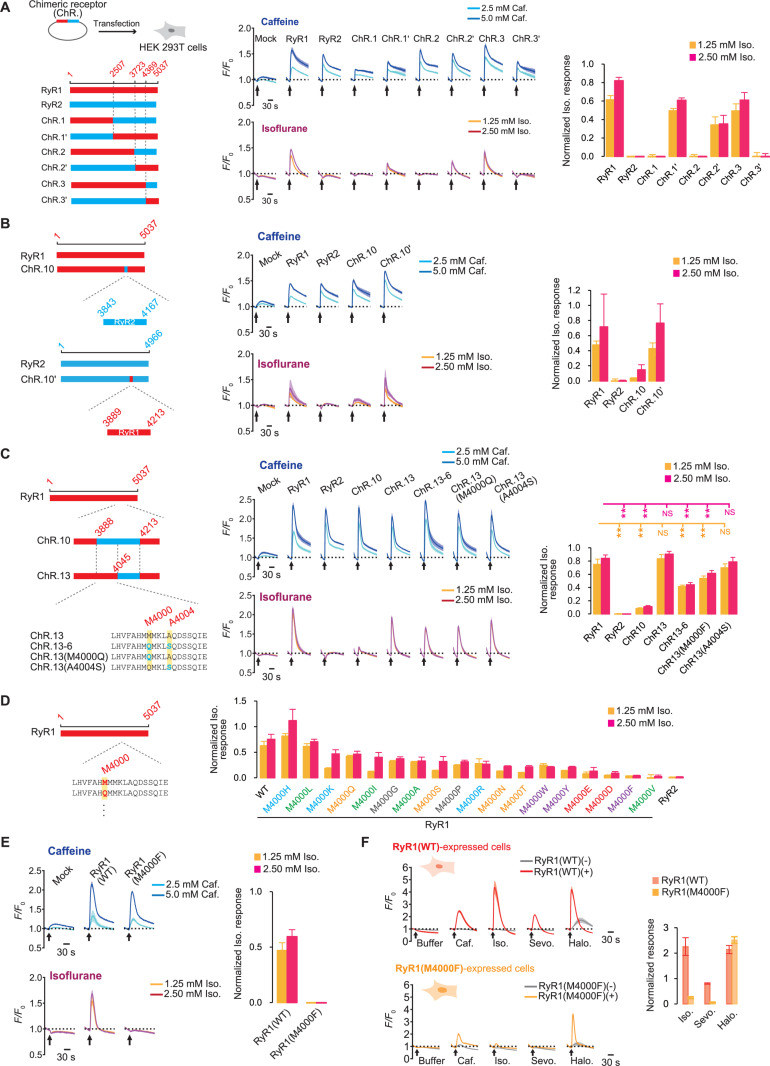
Identification of RyR1 residues responsible for the sensitivity to isoflurane. **(A)** Transient transfection of RyR1, RyR2, and the chimeric receptors (ChR.1-3, ChR.1′-3′) into HEK 293T cells. The time-course reaction (*F/F*_0_) and the peak of the isoflurane response normalized by the caffeine response are shown. *N* = 4. **(B)** The RyR1-RyR2 chimeric receptor 10 (ChR.10) and ChR.10′. *N* = 4. **(C)** The chimeric receptors, ChR.10, ChR.13, ChR.13-6, and the amino acid substitution mutants. *N* = 8. **(D)** Normalized isoflurane responses of M4000 mutants with single amino acid substitutions. The amino acid substitution was color-coded as follows: cyan for basic amino acids; red for acidic amino acids; orange for amino acids with polar uncharged side chains; green for amino acids with alkyl chains; and purple for amino acids with aromatic rings. *N* = 4–12. **(E)** Time-course reaction and normalized isoflurane response of RyR1(M4000F). *N* = 4. **(F)** Stable cell lines that express RyR1(WT) and RyR1(M4000F). Basal buffer without pharmacological agents, 1.25 mM caffeine, or 0.63 mM anesthetics (isoflurane, sevoflurane, and halothane) were given. *N* = 4. Data are represented as Mean ± SD. ** Adjusted *P*-values (Adj. *P*) < 0.01 by the Steel’s test (multiple comparisons between RyR1 and other groups). For the calculation of the Normalized Iso. Response (equation 1 in Materials and methods), the peaks at 5.0 mM caffeine and the control (without pharmacological agents) were normalized to 1.0 and 0, respectively, in the transient expression experiment **(A–E)**, while the peaks at 1.25 mM caffeine and the control were 1.0 and 0, respectively, in the experiments with stable cell lines **(F)**. ChR., Chimeric receptor; RyR1, the type 1 ryanodine receptor; RyR2, the type 2 ryanodine receptor; Caf., Caffeine; Iso., Isoflurane; Sevo., Sevoflurane; Halo., Halothane.

RyR1 and RyR2 proteins share a high degree of sequence identity, yet only RyR1 is sensitive to isoflurane. To pinpoint the RyR1 residues responsible for the response to isoflurane, we created a series of RyR1-RyR2 chimeric receptors (ChR.) (Fig 2A). Considering the availability of structural information [31,39–41], we opted to use the rabbit RyR1 sequence, while the mouse isoform of RyR2 was used. When transiently expressed in HEK 293T cells, both RyR1 and RyR2 produced a calcium response to caffeine. However, only cells expressing RyR1 were responsive to isoflurane, confirming that the selective action of isoflurane on RyR1 is reproduced using this transient expression method. To standardize the difference in the receptors’ responses to caffeine, data are normalized so that the peak intensity of the caffeine response is 1.0, while the peak intensity without any chemical treatment is 0 (Fig 2A right, equation 1 in Materials and methods). The chimeric receptor 1 (ChR.1), which incorporates the first half of the rabbit RyR1 sequence (RyR1^M1-D2507^) and the second half of the mouse RyR2 sequence (RyR2^R2473-N4966^), did not demonstrate sensitivity to isoflurane, although it could still be activated by caffeine (Fig 2A). Conversely, the reverse construct (ChR.1′) (which combines RyR2^M1-D2472^ and RyR1^R2508-S5037^) demonstrated isoflurane sensitivity, suggesting that the region responsible for the isoflurane response is located within the second half of the RyR1 sequence. Similarly, ChR.2, which comprises approximately the first ~3/4 portion of RyR1 and the remaining ~1/4 of RyR2, did not show isoflurane sensitivity. In contrast, ChR.2’ (constructed with the first ~3/4 portion as RyR2 and the remaining ~1/4 as RyR1) was sensitive to isoflurane. However, in the third series of chimeras, where the switch between RyR1 and RyR2 occurs around the first ~7/8 position, ChR.3 but not ChR.3′ responded to isoflurane. This suggests that the sequence difference between ChR.2 and ChR.3 (RyR1^A3724-G4369^, 646 amino acids) is responsible for the isoflurane response.

To examine the function of the sequence (RyR1^A3724-G4369^), we created ChR.4, in which this sequence was substituted with the corresponding sequence from RyR2 (RyR2^A3689-G4320^), along with the reverse chimeric construct ChR.4′ (S3A Fig). ChR.4 did not respond to isoflurane, whereas ChR.4′ did, indicating that the identified sequence (RyR1^A3724-G4369^) is both necessary and sufficient for the response. Although most of the subsequent chimeric receptors that were refined to focus on this region (ChR.5 to ChR.11) were activatable by isoflurane (S3B and S3C Fig), ChR.10 revealed a diminished response to isoflurane (S3C Fig). Notably, ChR.10 showed a normal response to caffeine (Fig 2B). The reverse construct (ChR.10′) which is based on RyR2 and includes the partial RyR1 sequence (RyR1^Q3889-S4213^) was activated by isoflurane in a manner comparable to wild-type RyR1 (Fig 2B). We concluded that these 325 amino acids (RyR1^Q3889-S4213^) are both necessary and sufficient for eliciting the response to isoflurane.

To further identify the specific amino acid residues responsible within the 325 amino acids region, we developed additional mutants, ChR.12 and ChR.13. In ChR.12, the upper half of the sequence is replaced with the corresponding RyR2 sequence, while in ChR.13, the latter half is substituted (S3D and S3E Fig). Unexpectedly, both ChR.12 and ChR.13 demonstrated isoflurane responses comparable to those of wild-type RyR1, indicating that this chimeric strategy is insufficient to effectively identify the specific residues responsible for isoflurane sensitivity. We then created a series of mutants, ChR.12-1 to ChR.12-9 or ChR.13-1 to ChR.13-8, where a portion of the latter (ChR.12-x) or upper (ChR.13-x) half of the sequence was replaced with RyR2 residues. ChR.12-4 and ChR.12-6 were excluded from the quantitative analysis due to their lack of response to caffeine. While ChR.12-2 was significantly more reactive to isoflurane than ChR.12, only ChR.13-6 was significantly less sensitive to isoflurane (S3D and S3E Fig). An independent set of experiments confirmed the reduced sensitivity (Fig 2C). We then considered whether the amino acids replaced in ChR.13-6, M4000 and/or A4004, are critical to the isoflurane response. The amino acid substitution mutant ChR.13(M4000Q) responded less than the wild-type RyR1, whereas ChR.13(A4004S) did not change significantly (Fig 2C). This suggests that the amino acid M4000 contributes more to the isoflurane sensitivity than A4004. The observation that the single amino acid substitution A4004 in wild-type RyR1 did not affect the isoflurane response supported A4004’s minor effect (S4A Fig).

To determine the significance of M4000 in the isoflurane response, we screened a series of mutants with single amino acid substitutions, replacing the M4000 with nearly all genetically codable amino acids (Fig 2D). RyR1(M4000Q), which was replaced with the corresponding RyR2 residue, was slightly less sensitive to isoflurane than RyR1(WT). In this substitution screening, amino acids with aromatic rings such as RyR1(M4000W), RyR1(M4000Y), and RyR1(M4000F) tended to be less sensitive to isoflurane, suggesting steric effects in specific binding forms. Furthermore, while mutants containing acidic amino acids (RyR1(M4000E) and RyR1(M4000D)) demonstrated reduced responses to isoflurane, RyR1(M4000V) had the greatest effect on isoflurane sensitivity. However, the caffeine response of M4000V was undetectable (S4B Fig), indicating that this mutation inhibits RyR1 activity. Then, we focused on the second-best mutant, RyR1(M4000F). An independent experiment confirmed that RyR1(M4000F) maintained the caffeine response but was nearly insensitive to isoflurane (Fig 2E). We created a HEK 293 cell line that expresses RyR1(M4000F) under the control of tetracycline. RyR1(M4000F)-expressed cells, like those in transient expression experiments, did not respond to isoflurane and sevoflurane at submillimolar concentrations, but they did respond to the caffeine (Figs 2F, S4C, and S4D).

Previous studies have identified the amino acid residues that participate in the binding of caffeine [31,42]. Based on these findings, we developed a series of caffeine-insensitive mutants. The majority of them, except for RyR1(I4996A), were nearly caffeine insensitive (S4E and S4F Fig). Among them, RyR1(W4716I), RyR1(W4716L), and RyR1(F3753A) were insensitive to caffeine but were activated by isoflurane. This suggests that the binding site of isoflurane is distinguishable from that of caffeine.

**Fig 3:**
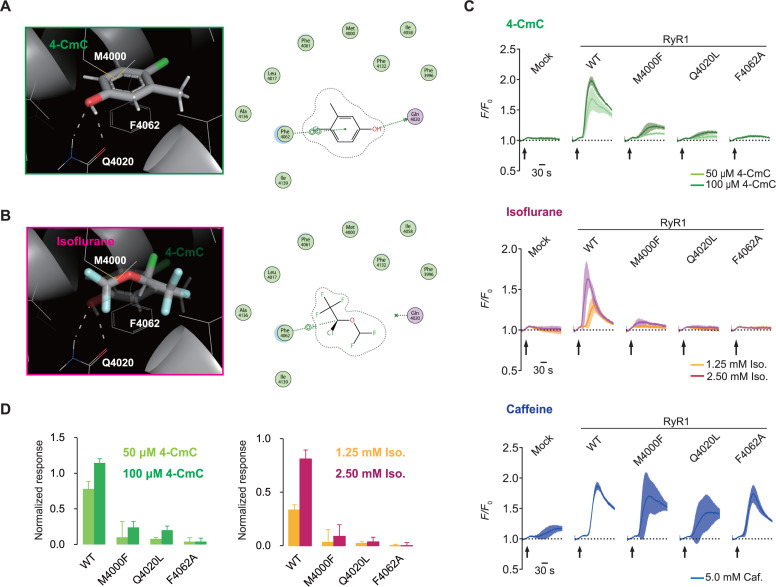
Putative isoflurane-binding site on RyR1. **(A, B)** Putative binding formation with 4-chloro-m-cresol (4-CmC) (A) and isoflurane (B). **(C)** Time-course reaction to 4-CmC, isoflurane, and caffeine of each amino acid substitution mutant. *N* = 4. **(D)** Normalized peak intensity of responses to 4-CmC and isoflurane for each amino acid substitution mutant. *N* = 4. The data are normalized so that the peaks of 5.0 mM caffeine and the control (without pharmacological agents) are 1.0 and 0, respectively (equation 1 in Materials and methods). Data are represented as Mean ± SD. 4-CmC, 4-chloro-m-cresol; RyR1, the type 1 ryanodine receptor; WT, wild-type; Caf., Caffeine; Iso., Isoflurane.

A recent study reported that the M4000 residue participates in the binding of 4-chloro-*m*-cresol (4-CmC) [43], an agonist of RyR1 and RyR2 [44–46]. By reconstituting the docking pose of 4-CmC and RyR1 based on the previous study (Fig 3A) [43], we were able to obtain a reasonable isoflurane binding form at the same site (Fig 3B). Consistent with a previous study [47], we found that the amino acid substitution mutant Q4020L had a reduced response to 4-CmC, and was also insensitive to isoflurane (Fig 3C and 3D). Furthermore, F4062 has the potential to contact 4-CmC through pi–pi stacking (Fig 3A) and interacts with isoflurane via a hydrogen bond (Fig 3B). We found that the substitution of phenylalanine with alanine (F4062A) substantially reduced the sensitivities to both 4-CmC and isoflurane (Fig 3C and 3D). These findings suggest that isoflurane activates RyR1 through a specific binding pocket, which is shared with the previously known agonist 4-CmC.

### Involvement of RyR1 in isoflurane anesthesia

**Fig 4:**
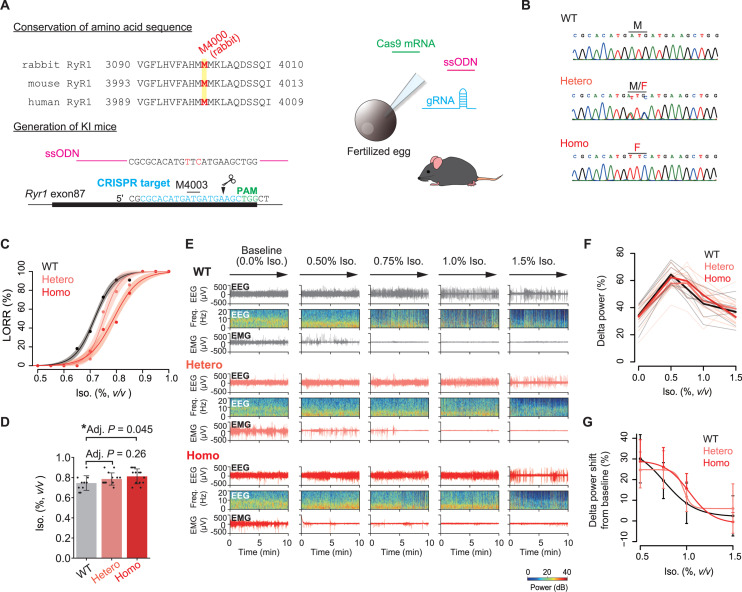
Resistance to isoflurane in RyR1(M4003F) KI mice. **(A)** Amino acid sequence around M4000 (rabbit RyR1) and the generation of RyR1(M4003F) KI mice. **(B)** The genomic sequences of the KI site for each genotype. **(C)** Dose–response curves for isoflurane-induced loss of righting reflex (LORR). Data were fitted with logistic functions (equation 5 in Materials and methods). *N* = 11 (WT), 14 (heterozygous [Hetero] KI), and 13 (homozygous [Homo] KI). The shaded regions represent 95% CIs. **(D)** Comparison of the concentrations at which the mice reached LORR (Mean ± SD with individual data points). *N* = 11 (WT), 14 (Hetero KI), and 13 (Homo KI). The adjusted *P*-values (Adj. *P*) by the Dunnett’s test are shown. **(E)** Representative transition of EEG signals, the spectrograms, and EMG signals during isoflurane treatment. At the baseline, mice were kept awake by gentle handling. **(F)** Normalized delta power (0.4–4 Hz) on EEG during isoflurane treatment for each animal. The mean values and individual data are shown. *N* = 8 (WT), 10 (Hetero KI), and 9 (Homo KI). **(G)** Changes in delta power over the baseline (0.0% isoflurane). Mean ± SD is shown. *N* = 8 (WT), 10 (Hetero KI), and 9 (Homo KI). Data were fitted using logistic functions (equation 6 in Materials and methods). RyR1, the type 1 ryanodine receptor; ssODN, single-stranded oligodeoxynucleotide; WT, wild-type; Hetero, heterozygous; Homo, homozygous; PAM, protospacer-adjacent motif; Iso., Isoflurane; LORR, loss of righting reflex; EEG, electroencephalogram; EMG, electromyogram; The mice illustration was modified from Openclipart (https://openclipart.org/).

The finding that RyR1 is activated by clinically relevant concentrations of isoflurane prompted us to investigate RyR1’s potential role in anesthetic effects. To examine this, we aim to generate mice with a modified endogenous RyR1 that is insensitive to isoflurane. The M4000 residue on rabbit RyR1, which is conserved across mammals, corresponds to M4003 of mouse RyR1 (Fig 4A). When expressed in HEK 293 cells, mouse RyR1(WT) was sensitive to isoflurane, as expected, whereas mouse RyR1(M4003F) was not activated by isoflurane while maintaining the caffeine response (S5A and S5B Fig). We then generated KI mice by mutating the endogenous RyR1 to M4003F using homology-directed repair with the CRISPR/Cas9 system and single-stranded oligodeoxynucleotide (ssODN) (Fig 4A and 4B) [48]. The heterozygous (Hetero) and homozygous (Homo) KI mice developed normally with no significant changes in body weight (S5C Fig). The KI mice also showed no observable behavioral changes. We quantitatively examined the sleep behavior of KI mice and found no significant differences in the basal and the homeostatic responses following sleep deprivation (SD) (S5D–S5I Fig).

We then assessed general anesthesia induced by isoflurane using the loss of righting reflex (LORR) as an indicator of unconsciousness [49]. For the LORR assay, sibling male animals for each genotype were used (*N* = 11 for WT mice, *N* = 14 for Hetero KI, *N* = 13 for Homo KI). The binary LORR results were plotted as the fraction of LORR animals against isoflurane concentrations (Fig 4C). In a fitted logistic curve, the EC_50_s of LORR were 0.72% (95% CI = 0.71–0.72) in WT, while 0.75% (95% CI = 0.74–0.76) in Hetero KI mice, and 0.79% (95% CI = 0.78–0.80) in Homo KI mice. The other variables are listed in the S1 Table. The EC_50_s of WT mice and Homo KI mice were significantly different (*P* < 0.0001 by *F*-test). When statistically comparing the isoflurane concentration required for LORR by multiple comparisons, Homo KI mice showed a significant increase compared to WT (*P* = 0.045 by the Dunnett’s test) (Fig 4D). To further evaluate this differential response, we recorded EEG and electromyogram (EMG) during isoflurane treatment (Fig 4E). At a hypnotic dose of isoflurane (0.50%), the EEG showed a significant increase in delta power (0.4–4 Hz) (Fig 4F). Consistent with a previous study [50], as isoflurane dosages were increased, the delta power gradually reduced with the occurrence of burst suppression (BS), an EEG signature of deep anesthesia in which the alpha activity (10–15 Hz) repeatedly emerges during flat EEG (Fig 4E and 4F) [51]. In the fitted logistic curve for the reduction of delta power (between 0.50% and 1.5% isoflurane, S1 Table), the IC_50_ values for each genotype were as follows: 0.75% (95% CI = 0.40–1.11) in WT mice, 0.97% (95% CI = 0.75–1.18) in Hetero KI mice, and 1.03% (95% CI = 0.93–1.12) in Homo KI mice. The IC_50_ in the Homo KI mice was significantly higher than that in the WT mice (*P* = 0.042 by *F*-test) (Fig 4G). Intriguingly, the concentration range for this differential delta power transition is close to the dose required to induce LORR. These findings suggest that RyR1 activation by isoflurane mediates general anesthesia at LORR-relevant concentrations.

### Inhibition of neuronal RyR1 alters the response to isoflurane

**Fig 5:**
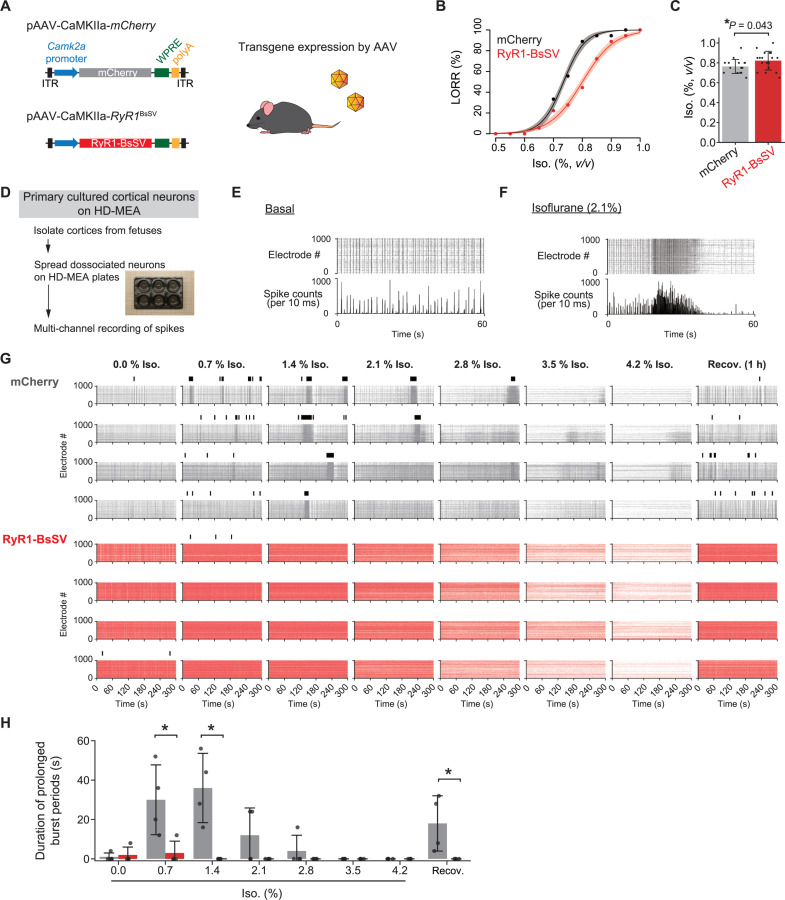
Inhibition of neuronal RyR1 alters the responses to isoflurane. **(A)** AAV-mediated expression in the brain. **(B)** Dose–response curves for loss of righting reflex (LORR) of mice expressing mCherry or RyR1-BsSV. Data were fitted with logistic functions (equation 5 in Materials and methods). *N* = 18 for each group. The shaded regions represent 95% CIs. **(C)** Comparison of the concentrations at which mice reach LORR (Mean ± SD with individual data points). *N* = 18 for each group. The *P*-value by the Student *t* test is shown. **(D)** Cultures of cortical neurons on HD-MEA. **(E, F)** Representative raster plots and the total spike counts in baseline (E) and the isoflurane treatment (2.1% isoflurane) (F). **(G)** Raster plots across the sequential exposure to isoflurane and the recovery for mCherry and RyR1-BsSV expressions. The above black bars represent the detected prolonged burst periods. Four independent wells are indicated, respectively. The extracellular calcium concentration was adjusted to 2.0 mM before the experiment. **(H)** Duration of the prolonged burst periods. Data are presented as Mean ± SD with individual data points. *N* = 4 for both mCherry (gray) and RyR1-BsSV (red) expressions. *P < 0.05 by the two-sample Wilcoxon test. Camk2a, Ca^2+^/calmodulin-dependent protein kinase II α; AAV, adeno-associated virus; RyR1-BsSV, brain-specific splicing variant of the type 1 ryanodine receptor; Iso., Isoflurane; LORR, loss of righting reflex; HD-MEA, high-density multielectrode array; Recov., Recovery. The mice illustration was modified from Openclipart (https://openclipart.org/).

Previous studies have shown that inhaled anesthetics induce calcium release from intracellular calcium stores in hippocampal and cerebral cortical neurons [52,53], which may be mediated by RyRs [52]. We then hypothesized that the neuronal calcium signal induced by inhaled anesthetics plays a role in anesthetic actions. RyR1-BsSV (an inhibitory form of RyR1) (S2E–S2H Fig) was expressed in the brain on a whole-brain scale using adeno-associated virus (AAV) (Fig 5A) [54,55]. While the mice expressing RyR1-BsSV showed no detectable changes in sleep behavior (S5J–S5N Fig), the EC_50_ in the fitted LORR curve of the RyR1-BsSV mice (0.80% [95% CI = 0.79–0.81]) was significantly higher than that of the mCherry control (0.73% [95% CI = 0.73–0.74], *P* < 0.0001 by *F*-test) (Fig 5B and S1 Table). A pairwise comparison of the concentrations required for LORR induction revealed significant resistance (*P* = 0.043 by the Student *t t*est) (Fig 5C). This suggests that perturbing neuronal RyR1 is sufficient to alter the animals’ responses to isoflurane.

In addition, to investigate the roles at a cellular level, we used primary cultures of cerebral cortical neurons. The electrical activity of cultured neurons was measured using a high-density multielectrode array (HD-MEA) (Fig 5D). Matured neurons cultured for more than a week showed synchronized bursts across the neuronal populations (Fig 5E). Isoflurane exposure reduced spikes and altered neuronal firing patterns (S6A and S6B Fig). Such effects of isoflurane were almost completely recovered after the removal of isoflurane from the medium, representing the reversibility of anesthetic actions. Interestingly, bursts were temporarily augmented and then suppressed on a time scale of tens of seconds during exposure to isoflurane (Figs 5F and S6A). This pattern (prolonged bursts) is reminiscent of BS, which occurs in EEG during deep anesthesia [51]. We found that the occurrence of the BS-like pattern is influenced by extracellular calcium concentration (S6A Fig), which is consistent with the previous findings that extracellular calcium is involved in BS switches in vivo [56,57].

We then used AAV to express RyR1-BsSV in the cortical neurons and assessed the response to isoflurane. We detected isoflurane-induced prolonged bursts by setting the threshold (see Materials and methods). In comparison to the mCherry-expressed control, the RyR1-BsSV-expressed condition showed almost no prolonged burst periods (Fig 5G and 5H). This suggests that inhibiting neuronal RyR1 blocks isoflurane-induced neuronal firing ensembles, lending support to RyR1’s involvement in the neuronal response to isoflurane.

### Identification of novel isoflurane-mimicking RyR1 agonists

**Fig 6:**
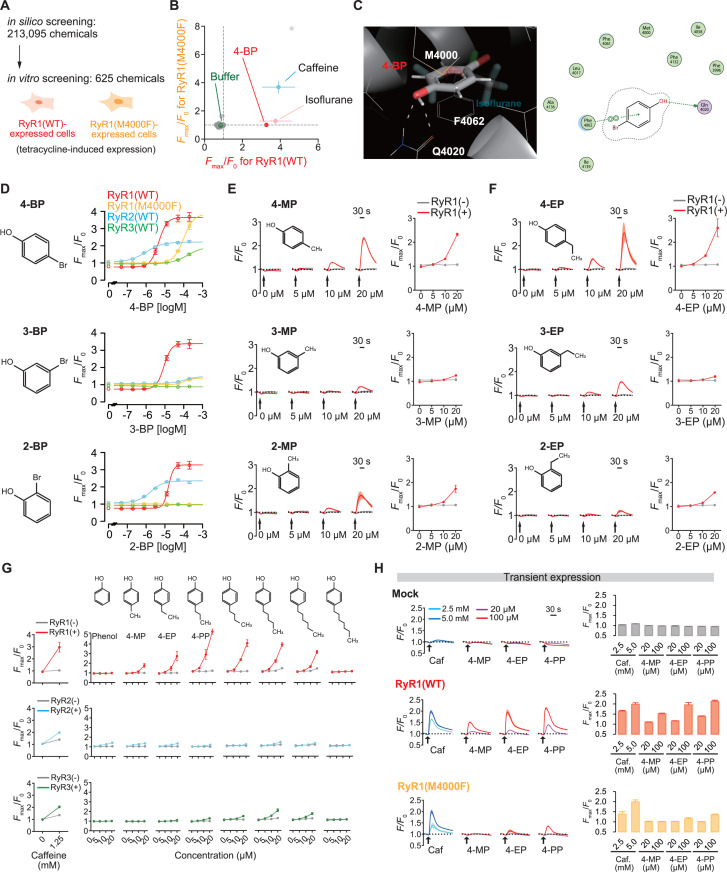
Identification of RyR1 agonists that share the binding site with isoflurane. **(A)** Scheme of the chemical screening. Following in silico screening, the agonistic actions of some of the hit compounds were tested with tetracycline-induced expressions of RyR1(WT) and RyR1(M4000F). **(B)** Results of the in vitro screening. As controls, stimulations by caffeine, isoflurane, and basal buffer without chemicals were included. 4-bromophenol (4-BP) is identified as a hit compound. **(C)** Putative binding formation of 4-BP. **(D)** Dose-dependent effects of 4-BP and the isomers 3-BP and 2-BP on RyR1(WT), RyR1(M4000F), RyR2, and RyR3. Data were fitted using logistic functions (equation 4 in Materials and methods). *N* = 4. (**E**, **F**) Effects of methyl phenols (MPs) (E) and ethyl phenols (EPs) (F) on RyR1. *N* = 5. **(G)** Effects of a series of 4-alkylphenols on RyR1, RyR2, and RyR3. *N* = 5. **(H)** Effects of 4-MP, 4-EP, and 4-PP on RyR1(WT) and RyR1(M4000F). The time-course reactions and the peak intensities are shown, respectively. *N* = 4. Data are presented as Mean ± SD. RyR1 and the M4000F mutant were expressed through tetracycline induction in the stable cell lines except for the data shown in panel **H**. RyR1, the type 1 ryanodine receptor; *F*_max_/*F*_0_, the maximum fluorescence intensity; 4-BP, 4-bromophenol; RyR2, the type 2 ryanodine receptor; RyR3, the type 3 ryanodine receptor; 4-BP, 4-bromophenol; 3-BP, 3-bromophenol; 2-BP, 2-bromophenol; 4-MP, 4-methylphenol; 3-MP, 3-methylphenol; 2-MP, 2-methylphenol; 4-EP, 4-ethylphenol; 3-EP, 3-ethylphenol; 2-EP, 2-ethylphenol; 4-PP, 4-propylphenol; Caf., Caffeine.

To further demonstrate the druggability of the isoflurane-binding site, we aimed to screen novel agonists. We conducted a large-scale in silico docking screening with a library of 213,095 chemicals (Fig 6A). Among the hit chemicals, we examined the effects of available chemicals on RyR1(WT) and RyR1(M4000F) using the tetracycline-induced expression system, as described in Fig 1. This screening revealed that 4-bromophenol (4-BP) activates RyR1(WT) but not RyR1(M4000F), similar to isoflurane (Fig 6B). 4-BP is a phenol with a bromo residue that has structural similarities to 4-CmC and is thought to share the binding site (Fig 6C). This supports our finding that isoflurane activates RyR1 in a similar manner to phenolic chemicals like 4-CmC. 4-BP potently activates RyR1(WT) (EC_50_ = 4.7 μM, 95% CI = 4.1–5.3), while it also has agonistic effects on RyR2 (EC_50_ = 1.0 μM, 95% CI = 0.8–1.1) and RyR1(M4000F) at high doses (Fig 6D). We found that the isomer 3-bromophenol (3-BP) has higher selectivity for RyR1(WT) than RyR1(M4000F), RyR2, or RyR3. While 4-BP and another isomer, 2-bromophenol (2-BP), target both RyR1 and RyR2 in the same way that 4-CmC does [46], 3-BP is almost completely selective for RyR1. 3-bromo-5-methoxyphenol (3-B-5-M), a structurally relevant chemical of 3-BP, did not activate RyR1 (S7A Fig), implying that RyR1 activation requires specific chemical structural properties.

We found that 4-methylphenol (4-MP) and 4-ethylphenol (4-EP), phenolic chemicals with a methyl or ethyl residue instead of the bromo residue, also have agonistic effects on RyR1 (Figs 6E, 6F and S7B). 4-alkylphenols show a structure-activity relationship with the cutoff effect of the carbon number [4,58], with 4-propylphenol (4-PP) as the top (Fig 6G), although the selectivity for RyR1(M4000F) is preserved in 4-MP but not in 4-EP and 4-PP (Fig 6H). Further analysis showed that the agonistic effects of the catechol derivatives are undetectable (S7C Fig), indicating that the phenolic structures are crucial for RyR1 activation [59]. Interestingly, 4-MP and 4-EP are biologically relevant chemicals that are metabolized by the gut microbiota, enter the blood, and potentially affect the activity of the central nervous system [60]. In the mammalian body, 4-MP and 4-EP are metabolized into 4-methylphenyl sulfate (4-MPS) and 4-ethylphenyl sulfate (4-EPS), respectively, by endogenous sulfotransferase [61,62], while neither 4-MPS nor 4-EPS activated RyR1 in our observation (S7D Fig).

### Novel isoflurane-mimicking RyR1 agonists induce a sedation-like state

**Fig 7:**
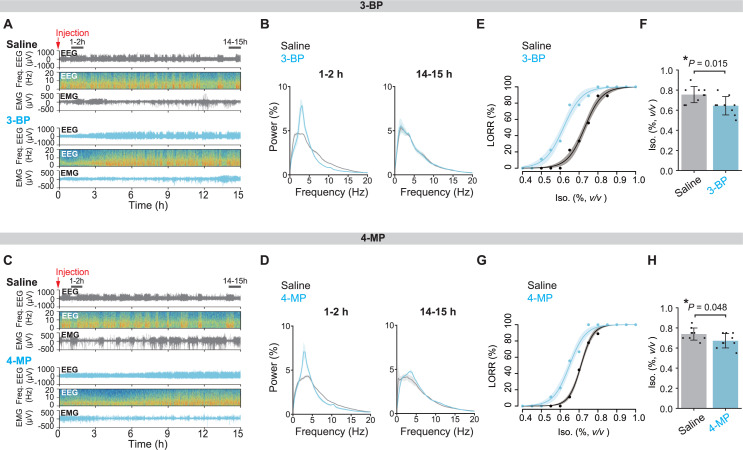
Isoflurane-mimicking RyR1 agonists induce a sedation-like state. **(A, B)** Representative transitions of EEG and EMG signals (A) and EEG power spectra (B) following the injection of 3-BP or saline as the control. *N* = 3 for both groups. In panel **B**, the line represents the mean value, and the shaded regions indicate the SEM. **(C, D)** Representative transitions of EEG and EMG signals (C) and EEG power spectra (Mean ± SEM) (D), following the injection of 4-MP or saline as the control. *N* = 3 for both groups. In panel **D**, the line represents the mean value, and the shaded regions show the SEM. **(E)** Dose–response curves of the loss of righting reflex (LORR) by isoflurane after saline or 3-BP. *N* = 9 for both groups. The shaded regions show 95% CIs. **(F)** Comparison of the concentrations at which mice reach LORR (Mean ± SD with individual data points). *N* = 9 for both groups. **(G)** LORR dose–response curves with isoflurane after saline or 4-MP injection. *N* = 9 for both groups. The shaded regions represent 95% CIs. **(H)** Comparing the concentrations at which mice reached LORR (Mean ± SD with individual data points). *N* = 9 for both groups. The data in panels **E** and **G** were fitted with logistic functions (equation 5 in Materials and methods). The *P*-value by the Student *t* test is shown in panels **F** and **H**. 3-BP, 3-bromophenol; 4-MP, 4-methylphenol; EEG, electroencephalogram; EMG, electromyogram; Iso., Isoflurane; LORR, loss of righting reflex.

We then assessed the in vivo relevance of RyR1 agonism by these newly identified chemicals. Upon systemic administration of 3-BP, the mice reduced spontaneous locomotor activity while maintaining their posture without exhibiting LORR, though behavioral changes were not formally quantified. The EEG showed a peak at low frequency (around 3–4 Hz) one hour after the injection, suggesting that the mice exhibited a sedation-like state (Fig 7A and 7B). After 14 h, the EEG pattern returned to a state similar to that of the control administered saline. 4-MP also showed a similar sedative-like effect (Fig 7C and 7D). Additionally, the preadministration of 3-BP reduced the isoflurane concentration required to induce LORR (Fig 7E and 7F). The EC_50_ of the fitted LORR curve for the 3-BP-administered group (0.62% [95% CI = 0.60–0.63]) was significantly lower than that of the saline-administered control (0.73% [95% CI = 0.72–0.74], *P* < 0.0001 by *F*-test) (Fig 7E and S1 Table). This difference was also supported by pairwise comparisons of the isoflurane concentrations needed to induce LORR (*P* = 0.015 by the Student *t t*est) (Fig 7F). The 4-MP administration resulted in a left shift in the LORR curve (EC_50_ of saline control: 0.71% [95% CI = 0.71–0.71], EC_50_ of 4-MP treatment: 0.65% [95% CI = 0.64–0.66], *P* < 0.0001 by *F*-test) (Fig 7G and 7H and S1 Table). These results suggest that the newly discovered RyR1 agonists enhance the sensitivity to isoflurane-induced LORR.

## Discussion

### RyR1 as a target of isoflurane

In this study, we showed that isoflurane and other inhaled anesthetics activate RyR1 (Fig 1) and discovered that the M4000 residue is critical for the response (Fig 2), which led to the identification of the putative binding pose (Fig 3). Previous research has identified GABA_A_Rs and K2P channels as direct targets of inhaled anesthetics involved in anesthetic actions [6,20], although inhaled anesthetics may affect a wide range of proteins. This study identified RyR1 as an additional substrate of inhaled anesthetics that is functionally related to their anesthetic/sedative actions.

We identified M4000 as a key residue responsible for RyR1’s response to isoflurane, and substituting phenylalanine (M4000F) significantly reduced sensitivity. The involvement of M4000 led us to consider that isoflurane shares the binding site with a previously known agonist, 4-CmC. It has been demonstrated that the specific amino acid sequence RyR1^S4007-R4180^ is required for the response to 4-CmC [63]. This sequence completely overlapped with the one found in this study (RyR1^Q3889-S4213^), which is necessary and sufficient for the response to isoflurane. We also demonstrated that mutants with reduced responses to 4-CmC, such as Q4020L [47] and F4062A, were not activated by isoflurane (Fig 3C and 3D), indicating that key residues are shared between them. 4-CmC, like caffeine [42,64], appears to activate RyR1 by increasing the calcium sensitivity of the channel [47]. It is reasonable to assume that inhaled anesthetics also do not solely open RyR1 channels; rather, they affect CICR by sensitizing the response to calcium. This is consistent with previous observations that halothane-induced calcium release in SR is dependent on calcium concentrations in the cytoplasmic side [65,66].

### Involvement of RyR1 in anesthetic/sedative actions

RyR1 has been recognized as a mediator of MHS. MH is a pharmacogenetic disorder of the skeletal muscle caused by inhaled anesthetics treatment, in which patients show hypermetabolic responses in the skeletal muscle. However, a previous study reported that MHS pigs have abnormal EEG at the onset of MH [67]. Another recent study reported that MHS mice exhibit deep anesthesia signature more quickly than non-MHS mice, even before showing severe MH symptoms [25]. These observations imply a potential role for RyR1 in anesthetic actions in the nervous system, though the causality is unclear.

In this study, we found that RyR1(M4003F) KI mice were resistant to isoflurane-induced LORR. The RyR1(M4003F) KI mice did not lose all sensitivity to isoflurane but rather showed a 10% increase in the EC_50_ of the LORR curve. A previous study found that mice with the KI mutation in the α1 subunit of GABA_A_Rs, which eliminates sensitivity to isoflurane, increased the EC_50_ of isoflurane-induced LORR by 14% [10]. For K2P channels, TASK-3 (KCNK9) knockout increased the EC_50_s of halothane- and isoflurane-induced LORR by 19%–38% and 5%, respectively [18,19]. In the case of TASK-1 (KCNK3), the KO mice showed 15%–20% and 6% increases in LORR EC_50_s under halothane and isoflurane anesthesia [18,19]. Overall, the isoflurane sensitivity effects of the KI mutations in GABA_A_Rs, K2P channels, and RyR1 are comparable in magnitude. Additionally, a previous study on a *Drosophila Ryr* ortholog found that transheterozygote flies with hypomorphic alleles have higher EC_50_s for the behavioral response to both halothane and isoflurane [26], suggesting that the RyR role in anesthetic action is evolutionarily conserved.

During isoflurane treatment, there was no detectable difference in delta power increase under the hypnotic state (at 0.5% isoflurane) and the deep anesthesia endpoint (at 1.5% isoflurane) (Fig 4E–4G). However, the transition of delta power from the hypnotic state to deep anesthesia was shifted to the right in Homo KI mice when compared to WT mice. This suggests that RyR1 is not primarily involved in hypnotic action but rather in the induction of LORR and the transition to deep anesthesia.

In this study, we also screened novel RyR1 agonists that shared the binding site with isoflurane and investigated the structure’s requirements, demonstrating that the isoflurane binding site is potentially druggable. We showed that the screened compounds induced a sedation-like state and sensitized mice to the effect of isoflurane. As a result, we anticipate that the 3-BP and isoflurane binding site of RyR1 would provide insights into the future development of clinically valuable chemicals. However, there are several limitations to directly applying the study’s findings to drug development. Firstly, we do not rule out the possibility that these compounds have multiple molecular targets, as inhaled anesthetics do. Furthermore, we did not assess long-term side effects, which limits further development for clinical use. The screened compounds (3-BP and 4-MP) are phenolic compounds. In the development of propofol, an injectable anesthetic, the anesthetic actions of alkylphenols were extensively screened using mice and rabbits, and most chemicals showed low therapeutic ratios (*i.e.*, hypnotic dosages were close to toxic dosages), except for promising hit compounds [68].

Given that inhibiting neuronal RyR1 leads to resistance to LORR (Fig 5), RyR1-dependent anesthetic action primarily derives from actions in neuronal tissues. Interestingly, in hippocampal neurons, isoflurane affects presynaptic calcium handling, and the effect is more pronounced in neurons derived from an MHS mouse model [53], implying a potential link to an anesthetic presynaptic mechanism [21]. The alternative or concurrent possibility is that RyR1 activation stimulates the calcium-dependent sleep regulatory pathway, including calcium-activated kinases like Ca^2+^/calmodulin-dependent protein kinase II (CaMKII) [55,69,70]. Given their similarities, sleep and general anesthesia may share some molecular mechanisms. Additionally, it is possible that RyR1-dependent calcium release selectively occurs in specific neurons. Future research will be required to determine the tissue/cell type-specific calcium release induced by inhaled anesthetics as well as the mechanism by which the calcium release is linked to anesthetic actions.

#### Materials and methods

##### Plasmids

For the expression of RyRs, pcDNA5/FRT/TO-rabbit *Ryr1* [71] and pcDNA5/FRT/TO-mouse *Ryr2* [72], which both express genes under the CMV promoter. To construct chimeric receptors (ChR.1-3 and ChR.1′-3′), appropriate sequences were amplified by polymerase chain reaction (PCR) from pcDNA5/FRT/TO-rabbit *Ryr1* or pcDNA5/FRT/TO-mouse *Ryr2* and ligated with the In-Fusion HD Cloning Kit (Takara Bio) according to the manufacturer’s instructions. For the remaining chimeric receptors and single amino acid substitution mutants, partial sequences of pcDNA5/FRT/TO-rabbit *Ryr1* or pcDNA5/FRT/TO-mouse *Ryr2* were subcloned into the pUC118 vector (Takara Bio), and mutagenesis was carried out accordingly. For the I4996A and Y5014A mutants of RyR1, a PCR-amplified sequence containing the coding sequence of RyR1^F4789-S5037^ and the stop codon (approximately 1.2 kbp) was cloned into the pUC118 vector using the Mighty Cloning Kit (Blunt End) (Takara Bio). After introducing the mutations through inverse PCR with the Mighty Cloning Reagent Set (Blunt End), the resulting pUC118 vectors were digested with a pair of restriction enzymes, ClaI and EcoRV (New England Biolabs), and cloned back into the pcDNA5/FRT/TO-rabbit *Ryr1*. For ChR.4′ and ChR.10′, a sequence containing the coding sequence of RyR2^E3335-N4966^ and the stop codon (approximately 5.0 kbp) was cloned into the pUC118 vector. The appropriate regions were replaced with the corresponding RyR1 sequences (RyR1^A3724-G4369^ for ChR.4′ and RyR1^Q3889-S4213^ for ChR.10′) using the In-Fusion HD Cloning Kit and cloned back into the pcDNA5/FRT/TO-mouse *Ryr2* with BstI and PmeI digestion (New England Biolabs). Otherwise, the pUC118 vector containing the coding sequence of RyR1^E3212-L4788^ (approximately 4.7 kbp) was used with AvrII and ClaI digestion (New England Biolabs). To express and generate stable cell lines of mouse *Ryr1*, the cDNA was subcloned into the pcDNA5/FRT/TO vector. For RyR1-BsSV (pcDNA5/FRT/TO-*Ryr1*^BsSV^), the carboxyl-terminal portion encoding RyR1^M4382-S5037^ (656 amino acids) [34,35] was amplified by inverse PCR from pcDNA5/FRT/TO-rabbit *Ryr1* and ligated with the DNA Ligation Kit (Mighty Mix) (Takara Bio). For pcDNA5/FRT/TO-*mCherry*, the coding sequence of pcDNA5/FRT/TO-*Ryr1*^BsSV^ was replaced with a mCherry encoding sequence using the In-Fusion HD Cloning Kit (Takara Bio). For the construction of pAAV plasmids (pAAV-CaMKIIa-*RyR1*^BsSV^ and pAAV-CaMKIIa-*mCherry*), pAAV-hSyn-DIO-hM3D(Gq)-mCherry (a gift from Dr. Bryan Roth, Addgene plasmid # 44,361; http://n2t.net/addgene:44361; RRID: Addgene_44361) [73] was used as the template. The promoter region was replaced with the *Camk2a* promoter. Furthermore, the DIO-hM3D(Gq)-mCherry was replaced with RyR1-BsSV or mCherry using In-Fusion HD Cloning Kit (Takara Bio) after SalI and EcoRI digestion. For the expression of 5ppase, pCIS-CAG-tdTomato-*5ppase*^WT^ and pCIS-CAG-tdTomato-*5ppase*^R343A/R350A^ were used.

##### Stable HEK 293 cell lines for the expression of RyRs

The tetracycline-inducible stable HEK 293 cell lines were derived from the Flp-In T-Rex system [27] and were used to express RyR1, RyR2, and RyR3. The sequences of each RyR isoform were derived from rabbits. The cell lines were maintained in a culture medium containing Dulbecco’s Modified Eagle Medium (DMEM) (high glucose, pyruvate, Thermo Fisher Scientific), 10% (*v/v*) fetal bovine serum (FBS) (Sigma-Aldrich), and 100 U/ml penicillin-streptomycin (Thermo Fisher Scientific) at 37 °C and 5% CO_2_. To develop stable lines for rabbit RyR1(M4000F), mouse RyR1(WT), and mouse RyR1(M4003F), pcDNA5/FRT/TO-rabbit *Ryr1*^M4000F^, pcDNA5/FRT/TO-mouse *Ryr1*^WT^, or pcDNA5/FRT/TO-mouse *Ryr1*^M4003F^ was transfected into Flp-In T-Rex HEK 293 cells (Invitrogen) with the pOG44 vector encoding Flp recombinase, according to the manufacturer’s instructions. The cells were selected using 10 μg/ml Blasticidin S Hydrochloride (Wako Pure Chemical) and 200 μg/ml Hygromycin B (Nacalai Tesque). To induce expression for assays, the cells were seeded in poly-d-lysine (PDL)-coated 384-well plates (Corning) at a density of 20,000–50,000 cells/well. The culture medium was supplemented with 20 μg/ml tetracycline hydrochloride (Sigma-Aldrich). The culture without tetracycline served as the control. The plates were incubated overnight at 37 °C with 5%.

##### Transfection of the plasmid DNA

HEK 293T cells were kept in the culture medium at 37 °C and 5% CO_2_. To transfect plasmids encoding RyR1, RyR2, chimeras, and the amino acid substitution mutants, the plasmid DNA solution was divided into each well of the PDL-coated 384-well plates (50 ng each), followed by adding the mixture of FuGENE6 Transfection Reagent (Promega) (150 nl) and the culture medium without FBS (20 μl). After 20 min of incubation at room temperature, HEK 293T cells were added at a density of 15,000 cells/well with FBS at the final concentration of 10% (*v/v*) and incubated for two days. For heterologous expression of RyR1(WT) and RyR1-BsSV (or mCherry), pcDNA5/FRT/TO-*Ryr1*^BsSV^ or pcDNA5/FRT/TO-*mCherry* was transfected into the tetracycline-inducible stable cell line of RyR1 using nearly the same protocol, but with 750 nl of the FuGENE6 Transfection Reagent for each well. For the heterologous expression of RyR1(WT) and 5ppase, the expression vectors of 5ppase were transfected into the stable line of RyR1 using 225 nl of the FuGENE6 Transfection Reagent.

##### Measurement of the intracellular calcium

After inducing RyR expression in 384-well plates (see above), the medium was replaced with Hanks’ Balanced Salt Solution (HBSS) (Sigma-Aldrich), which was supplemented with 20 mM 2-[4-(2-Hydroxyethyl)-1-piperazinyl]ethanesulfonic acid (Nacalai Tesque), 2.5–10 μM Cal-520 AM (AAT Bioquest), and 0.04% (*w/w*) F-127 (Sigma-Aldrich), and adjusted to pH 7.3 by sodium hydroxide. After 90 min of incubation at 37 °C in 5% CO_2_, Allura red AC (TCI) was added as an extracellular fluorescence quencher at a final concentration of 6.25 mM. The plates were placed into a functional drug screening system FDSS7000 (Hamamatsu) and the fluorescence signal was measured at 1-s intervals (excitation at 480 nm, emission at 540 nm, exposure time: 200 ms). The experiments were carried out at room temperature. Following a baseline measurement of 10 or 30 s, pharmacological agents were automatically and simultaneously applied to each well. The baseline fluorescence intensity (*F*_0_) was calculated as the average intensity over the first 10 or 30 s. Then, the relative fluorescence level (*F*/*F*_0_) and the peak intensity (*F*_max_/*F*_0_) were determined. To compare different RyR isoforms or mutants, the *F*_max_/*F*_0_ values were normalized so that the mean values of caffeine stimulation and the control without pharmacological agents were 1.0 and 0, respectively, as shown below:


$$\begin{array}{c} Normalized\;response=\;\frac{F_{\max}{/F}_{0}\;-\;{MEAN}_{control}}{{MEAN}_{caf}-\;{MEAN}_{control}} \end{array}$$ (1)

where *MEAN*_control_ and *MEAN*_caf_ represent the mean of *F*_max_/*F*_0_ values for the control (no pharmacological agents) and caffeine administration, respectively. For the dantrolene treatment, given the temperature-dependent action, cells were adequately incubated at 37 °C before the assay with dantrolene sodium salt (Sigma-Aldrich). Ca^2+^/Mg^2+^-free HBSS (Sigma-Aldrich) (adjusted to pH 7.3) was used for the nominal calcium-free condition and the TG treatment, with the addition of 1 mM ethylene glycol tetraacetic acid (EGTA, Nacalai Tesque). For the control, the Ca^2+^/Mg^2+^-free HBSS was supplemented with 1.26 mM calcium chloride. In the TG treatment, 2 μM TG (Nacalai Tesque) was dissolved in the Ca^2+^/Mg^2+^-free HBSS containing EGTA.

##### Docking simulation of 4-CmC and isoflurane to RyR1

The docking of 4-CmC and isoflurane to potential RyR1 binding sites was carried out in the following steps: (1) structure preparation of small molecules and RyR1 for docking, (2) docking of the 4-CmC molecule using the model from the previous study [43], and (3) docking of isoflurane using the 4-CmC/RyR1 docking model. The initial structure of human RyR1 (Q3889–S4213) was modeled based on rabbit RyR1 chain E (PDB: 5TAL) [31]. This domain contains identical amino acid sequences for human RyR1 and rabbit RyR1. The human RyR1 structure was refined for docking simulations using the Protein Preparation Wizard [74] Script within Maestro (Schrödinger, LLC). For small molecules, ionization and energy minimization were performed by the OPLS3 force field in the LigPrep Script in the Maestro (Schrödinger, LLC). These minimized structures served as input structures for docking simulations. Next, the putative binding site on the 4-CmC/RyR1 complex was estimated using the model diagram from the previous study [43]. The docking simulation of 4-CmC was carried out using the Induced Fit Docking [75] program (Schrödinger, LLC). The flexible residue M4000 was specified with the Trim side chain option. The Rank1 model of the best 20 poses was selected as the final model of the 4-CmC/RyR1 complex based on the IFD score. The docking simulation of isoflurane was conducted using the Glide [76,77] SP docking program (Schrödinger, LLC). Up to 10 docking poses for the isoflurane molecule were generated in a grid box defined by the 4-CmC binding site position from the previous step. The best model of isoflurane binding was selected based on the Glide score. The structural models in the figures were depicted using PyMOL version 2.5.4 software (Schrödinger, LLC).

##### Chemical screening

We carried out in silico library screening based on the 4-CmC binding site on the RyR1 complex from the previous step, using molecular docking with the Glide SP model against 213,095 compounds at the Drug Discovery Initiative, Graduate School of Pharmaceutical Sciences, The University of Tokyo (https://www.ddi.f.u-tokyo.ac.jp/en/). For all compounds in the chemical library, ionization and energy minimization were performed by the OPLS3 force field in the LigPrep Script in the Maestro (Schrödinger, LLC). In vitro screening was performed using tetracycline-inducible stable cell lines of rabbit RyR1(WT) and rabbit RyR1(M4000F). The 384-well culture plates were prepared as described above (100,000 cells/well). Each chemical was dissolved in dimethyl sulfoxide (DMSO) and used at a final concentration of 10 μM. The final concentration of DMSO was 0.05% (*v/v*). Stimulations by caffeine, isoflurane, and a nonchemical control were included in the same plates.

##### Animals

All experimental procedures and housing conditions were approved by the Institutional Animal Care and Use Committee of the University of Tokyo (Approve numbers: M-P17-129, M-P22-097, A2024M080). The University of Tokyo is approved as Animal Welfare Assurance number: F18-00412 (NIH OLAW). All animals were cared for in accordance with the Institutional Guidelines for Experiments using Animals. All mice had *ad libitum* access to food and water and were kept at ambient temperature and humidity using a 12-h light/12-h dark cycle. C57BL/6N mice (CLEA Japan) were used for the behavioral experiments, and ICR mice (SLC Japan) were used to prepare primary cultures of cortical neurons. The mice used in each experiment were chosen at random from the colonies.

##### Generation of KI mice

The M4003 residue in the endogenous *Ryr1* locus (NM_009109) was mutated by homology-directed repair with CRISPR/Cas9-mediated KI using ssODN [48]. The preparation of gRNA and Cas9 followed the previous study [55]. The ssODN sequence and primer sequences used for producing the gRNA are listed in S2 Table. Approximately 1–2 pl of RNase-free water (Nacalai Tesque) with 100 ng/μl gRNA, 100 ng/μl Cas9 mRNA, and 100 ng/μl ssODN was microinjected into the cytoplasm of fertilized eggs in M2 medium (Merck, ARK Resource). The embryos were then cultured for 1 h in KSOM medium (Merck, ARK Resource) with 5% CO_2_ at 37 °C. Approximately 20 embryos were transferred into the oviducts of pseudopregnant female ICR mice. Genomic DNA was extracted from the tails using the DNeasy Blood and Tissue Kit (QIAGEN) according to the manufacturer’s instructions. To select the KI mice, genomic DNA was PCR-amplified using the appropriate primer pair (S2 Table), and the amplicons were sequenced. The obtained KI mice were crossed with WT mice and kept as heterozygous mice for several generations. For the experiments, heterozygous mice were crossed, and their male siblings were used.

##### Sleep measurement

Sleep was measured using the Snappy Sleep Stager (SSS), a fully automated and noninvasive respiration-based sleep phenotyping system [78]. The recording and analysis protocols were based on the original study. Basically, 8- to 11-week-old mice were placed in SSS chambers for a week to record sleep. Throughout the recording, the mice were kept under a 12-h light/12-h dark cycle and had *ad libitum* access to food and water. Data from the first day (Day 1) were excluded from the analysis. Sleep staging was performed every 8-s epoch. Sleep parameters, such as sleep duration, *P*_WS_ (transition probability from wakefulness to sleep), *P*_SW_ (transition probability from sleep to wakefulness), and sleep episode duration, were calculated. Sleep duration was calculated using the number of epochs annotated as sleep. For the transition probabilities, there are four types of state transitions between two consecutive epochs: wakefulness to sleep, sleep to wakefulness, sleep to sleep, and wakefulness to wakefulness. The transition probabilities were then calculated using all two consecutive epochs as follows:


$$\begin{array}{c} P_{ws}=\;\frac{N_{ws}}{N_{ws}+\;N_{ww}}\; \end{array}$$ (2)

$$\begin{array}{c} P_{sw}=\;\frac{N_{sw}}{N_{sw}+\;N_{ss}}\; \end{array}$$ (3)

where *N*_MN_ represents the number of transitions from state *M* to state *N*, and *W* and *S* denote wakefulness and sleep, respectively. The sleep episode duration is the average amount of time spent in each sleep bout. For the SD experiment, 24-week-old KI mice and their sibling WT mice were used. The sleep recordings were made for 2 weeks. Baseline sleep was recorded in the first week. SD was carried out on Day 6 of the second week. For SD, mice were placed on a shaker for 6 h (zeitgeber time 0–6 [ZT0-ZT6]). The mice were then returned to the recording chambers, and the subsequent sleep was measured.

##### Evaluation of loss of righting reflex (LORR)

The LORR analysis was carried out based on previous studies with some modifications [16,49]. 8- to 10-week-old mice were used for experiments. For exposure to isoflurane, mice were placed in gas-tight cylindrical chambers. After a 15-min habituation period, isoflurane gas was introduced with a vaporizer at a rate of 1.0 l/min and stepwise increased from 0.40% (*v/v*) to 1.0% at a 0.05% pitch. The isoflurane concentration was continuously monitored using a concentration meter (RIKAKEN KEIKI). At each step, after 5 min of equilibrium, the righting reflex was assessed by turning the mouse supine. A mouse was considered to have lost its righting reflex if it did not turn with at least three paws within 30 seconds. The chambers were warmed to 37 °C throughout the assay. Experiments were conducted between ZT6 and ZT12. The genotypes of the KI mice were determined after the experiments so that the experimenters were unaware of them. The novel RyR1 agonists were dissolved in saline, mixed thoroughly, and intraperitoneally injected at a dosage of 100 mg/kg one hour before the LORR test.

##### EEG/EMG recordings

KI mice aged 12–14 weeks and their sibling WT mice were used. The mice were implanted with EEG and EMG electrodes. Two stainless steel EEG recording screws with a diameter of 1.0 mm and a length of 2.0 mm were implanted on the skull of the cortex (anterior, +1.0 mm; right, +1.5 mm from bregma or lambda). EEG and EMG electrodes with 4 pins in a 2-mm pitch (Hirose Electric) and soldered wires were wrapped around the screws. EMG activity was monitored using stainless steel, Teflon-coated wires (diameter: 0.33 mm, Cooner Wire) connected to electrodes, and the other end placed into the trapezius muscle. Finally, the fixed electrodes were completely covered with dental cement (Unifast III, GC Corporation), and the mice’ scalps were sutured. After a 9- to 10-day recovery period, the mice were placed in experimental cages with a connection of spring-supported recording leads. The EEG/EMG signals were amplified (Nihon Kohden, Miyuki Giken), digitized at a sampling rate of 100 Hz, and recorded with VitalRecorder software (KISSEI Comtec). Experiments were carried out between ZT0 and ZT12. The mice were kept awake during the baseline recording (0.0% isoflurane) by gentle handling. During the isoflurane exposure, the mice were kept in a chamber. After more than 15 min of habituation, isoflurane gas was introduced into the vaporizer at a rate of 1.0 l/min. The isoflurane concentration was increased stepwise from 0.0% (*v/v*) to 0.5%, 0.75%, 1.0%, and 1.5%, with the mice being exposed to isoflurane for 10 min at each step. The isoflurane concentration was continuously monitored using the concentration meter. For the injection of novel RyR1 agonists, each chemical was administered at ZT4 as described above. The mice were given saline at ZT4 as a control the day before the chemical injection.

##### AAV packaging, purification, titration, and retro-orbital injection

AAV-PHP.eB was prepared based on a previous report with some modifications [79]. AAV pro 293T cells (Takara Bio) were cultured in 150-mm dishes in the above-mentioned culture medium at 37 °C with 5% CO_2_. The pAAV plasmid, pUCmini-iCAP-PHPeB (a gift from Dr. Viviana Gradinaru, Addgene plasmid # 103005; http://n2t.net/addgene:103005; RRID: Addgene_103005) [54], and the pHelper plasmid (Agilent) were transfected into the cells at >90% confluency with polyethyleneimine (PEI, Linear, MW 25,000, Polysciences). The pAAV:pUCmini-iCAP-PHPeB:pHelper ratio was optimized to 1:4:2 (DNA weight). After 24 h of incubation, the culture medium was replaced with a specialized medium containing DMEM (high glucose, GlutaMAX Supplement, pyruvate, Thermo Fisher Scientific), 5% (*v/v*) FBS, MEM nonessential amino acids solution (Thermo Fisher Scientific), and 100 U/ml penicillin-streptomycin. After 2 days, the medium was collected and replaced with the specialized medium. The collected medium was kept at 4 °C. After three more days, the cells were harvested together with the medium. The following procedures were performed on the assembly of the first and second collections. The suspension was separated into supernatant and cell pellet by centrifugation (2,000*g* for 15 min). The obtained pellets were suspended in a Tris-MgCl_2_ buffer (10 mM Tris and 2 mM MgCl_2_) and freeze-thawed three times in liquid nitrogen. The extract was then treated with TurboNuclease (250 U, Accelagen) for 1 h at 37 °C. Polyethylene glycol (PEG) (MW 8,000, MP Biomedicals) was added to the supernatant at a final concentration of 8% and ice-cooled for 2 h. Following centrifugation (4,000*g* for 30 min at 4 °C), the PEG mixture was suspended in the Tris-MgCl_2_ buffer, assembled with the pellet-derived extract, and incubated at 37 °C for 30 min. The extracts were then purified using ultracentrifugation (Optima E-90, Type 70 Ti rotor with 32.4 mL OptiSeal tubes, Beckman Coulter, 58,400 rpm for 145 min at 18 °C) with Iodixanol density gradients (15%, 25%, 40%, and 60% [*w/v*], OptiPrep, Serumwerk Bernburg). AAV localized in the 40% solution was collected and filtered using the Amicon Ultra-15 centrifugal filter (100 kDa, Merck), and eluted with D-PBS (Nacalai Tesque). To titrate AAV, the solution was treated with TurboNuclease (100 U) at 37 °C for 1 h, followed by the treatment with 0.25 mg/ml Proteinase K (Nacalai Tesque) at 37 °C for 1 h. The viral genomes were extracted by phenol: chloroform: isoamyl alcohol 25:24:1 (Nacalai Tesque), precipitated with isopropanol, and then dissolved in Tris-EDTA buffer. The titer was calculated using the number of WPRE sequences within the viral genome. Quantitative PCR (qPCR) was done using TB Green Premix Ex Taq GC (Takara Bio). The primer sets are shown in S2 Table. Following the initial denaturation at 95 °C for 60 s, a cycle of 95 °C for 10 s and 60 °C for 30 s was performed for cycles. Mice aged 6 weeks were anesthetized with isoflurane before receiving a 100 μl injection of AAV solution into their retro-orbital sinus. The mice were examined for sleep and LORR at 8 and 9 weeks of age, respectively. The AAV injection titer was 2.0 × 10^11^ vg/mouse for both pAAV-CaMKIIa-*mCherry* and pAAV-CaMKIIa-*RyR1*^BsSV^.

##### Primary culture of the cerebral cortex on high-density multielectrode array

The MaxTwo High-Density Microelectrode Array (HD-MEA) (MaxWell Biosystems) was used to record spikes from primary cultured cortical neurons. The HD-MEA plates were pre-coated according to the manufacturer’s instructions with minor modifications. After treatment with 1% (*w/v*) Terg-a-zyme solution (Sigma-Aldrich) for two days, the plates were washed three times and sanitized with 70% (*v/v*) ethanol for 15 min. After washing twice with sterile deionized water (SDW), the electrodes on the plates were coated with 0.07% PEI at 4 °C overnight. The PEI solution was made by diluting a 50% (*w/v*) solution in H_2_O (Mw 750,000, Sigma-Aldrich) with borate buffer (Thermo Fisher Scientific). After washing the electrodes three times with SDW, 1% (*v/v*) Geltrex (Thermo Fisher Scientific) diluted in DMEM (high glucose, Thermo Fisher Scientific) with 100 U/ml penicillin-streptomycin was applied (20 µl each). The plates were then incubated at 37 °C with 5% CO_2_ for 1 h. Cortical primary cells were isolated from ICR mice on embryonic day 16 (E16). Isolated cortices were collected in ice-cold DMEM containing 100 U/ml penicillin-streptomycin. Approximately 10 cortices were washed twice with the Ca^2+^/Mg^2+^-free HBSS before being treated with 200 µl of Neuron Isolation Enzyme with Papain (Thermo Fisher Scientific) for 30 min at 37 °C. Then, 2 ml of FBS and 1 mg of DNase I (Roche) were added. Following centrifugation (800 rpm for 3 min at 4 °C), the cell pellets were resuspended in DMEM containing 10% (*v/v*) FBS and 100 U/ml penicillin-streptomycin (the same as the culture medium described above). Cell suspensions were filtered through a 70 µm mesh (FALCON) and resuspended in the culture medium. After adjusting the cell number, the cell suspension (20 µl) was placed onto the electrodes at a final density of 150,000 cells/well. After 1 hour of incubation at 37 °C with 5% CO_2,_ 1.2 ml of the culture medium was added. The plates were then cultured at 37 °C with 5% CO_2_, and half of the medium was replaced with Neurobasal Plus Medium (Thermo Fisher Scientific) supplemented with B-27 Plus Supplement (Thermo Fisher Scientific) twice a week.

##### Recording of HD-MEA, treatment with anesthetics, and infection with AAV

A program (Activity Scan Assay) in MaxLab software (MaxWell Biosystems) selected active electrodes that were thought to be adjacent to firing neurons after culturing for 7–12 days in vitro (DIV7–12). The activity of the selected active electrodes (1,024 electrodes) was measured for analysis. Basically, the experiments were carried out on DIV13–14. For the isoflurane treatment, the plates were treated with vaporized isoflurane at 37 °C. The concentration was gradually increased from 0.0% (*v/v*) to 4.2% at 0.7% pitch. After 5 min of isoflurane exposure, the electrical signal was recorded for 5 min at each step. Throughout the recording, the temperature was kept at 37 °C with 5% CO_2_ circulation. To assess the recovery after isoflurane treatment, the plates were left for 1 h to allow the isoflurane to evaporate from the medium. To adjust extracellular calcium, EGTA (Sigma-Aldrich) was added to the medium at a final concentration of 2 mM, followed by calcium chloride supplementation. Spikes were identified as electrical signals that exceeded 5 times the standard deviation. To detect the prolonged burst periods caused by isoflurane exposure, the data were segmented into 4-s epochs, and the total firings in each epoch were determined. Epochs with firings greater than three times the standard deviation of the basal condition (0.0% isoflurane) were classified as prolonged burst periods, and the total length of the annotated epochs was calculated accordingly. For the AAV infection, primary cultured neurons were infected with AAV on DIV7 (multiplicity of infection: 1.3 × 10^6^). After removing the culture medium, the neurons were exposed to AAV solutions for 1 h at 37 °C with 5% CO_2_, followed by the addition of 1 ml of Neurobasal Plus Medium supplemented with B-27 Plus. After four days, the medium volume was increased to 2 ml and used in the experiments on DIV17 (10 days after the infection).

##### Statistics

Sample sizes were not determined by statistical methods. All statistical analyses were performed using R (version 4.3.2). The drc package of R was used in dose-response analyses [80]. For the dose-dependent RyRs’ response to agonists, the peak intensities (*F*_max_/*F*_0_, *P* in equation 4) were plotted against log[*drug*] and fitted to four-variable logistic functions using the drm function (within the drc package) as follows:


$$\begin{array}{c} P\;=P_{\min}+\frac{P_{\max}-P_{\min}}{1+\;exp(H(\log{EC}_{50}-log[drug]))} \end{array}$$ (4)

where *P*_max_ and *P*_min_ represent the maximum and minimum peak intensities, respectively, and *H* is the Hill slope. The EC_50_ represents the half-maximal effective concentration. To determine the dose-response of LORR, the binary results were plotted as the percentage of animals with LORR against drug concentrations. These were fitted to the following two-variable logistic functions (the drm function):


$$\begin{array}{c} LORR\;(\%)=\;\frac{100}{1+\;exp(H(\log{EC}_{50}-\log[drug]))}\; \end{array}$$ (5)

where *H* and EC_50_ denote the Hill slope and half-maximal effective concentration, respectively. To fit the delta power transitions, the delta power shift (*ΔD* in equation 6) was plotted against the drug concentration and fitted to the following four-variable logistic functions (the drm function):


$$\begin{array}{c} \Delta D=\Delta D_{\min}+\;\frac{\Delta D_{\max}-\Delta D_{\min}}{1+\;exp(H\left(\log[drug]-\log{IC}_{50}\right))}\; \end{array}$$ (6)

where *ΔD*_max_ and *ΔD*_min_ are the maximum and minimum *ΔD* values, respectively, and *H* is the Hill slope. The IC_50_ represents the half-maximal inhibitory concentration. For these dose-response analyses, the estimates of each variable and their standard errors were reported in S1 Table. 95% CIs of EC_50_ were also reported. The *F*-test was used to perform pairwise comparisons of parameters (e.g., EC_50_) from different fitted curves [80].

For the other statistical comparisons, the statistical methods were determined based on normality and homogeneity of variance. For two-sample tests, normality and homogeneity of variance were evaluated by the Shapiro test and the *F*-test, respectively (significance levels are 0.05 in both tests). For samples where normality and homogeneity of variance were satisfied (*P* ≥ 0.05 in both tests), Student *t* test was applied. When normality was satisfied (*P* ≥ 0.05 by Shapiro test) but homogeneity of variance was not (*P* < 0.05 by *F*-test), the Welch’s *t t*est was applied. Otherwise, the two-sample Wilcoxon test was applied. When comparing paired samples, the paired *t t*est was applied. For the multiple comparisons, normality and homogeneity of variance were evaluated using the Kolmogorov-Smirnov test and the Bartlett test, respectively (significance levels are 0.05 in both tests). For the comparisons against a single identical control, the Dunnett’s test was applied when normality and homogeneity of variance were met (*P* ≥ 0.05 in both tests). Otherwise, Steel’s test was used. For multiple comparisons with each combination, the Tukey–Kramer test was used on samples with normality and homogeneity of variance. Otherwise, the Steel-Dwass test was applied. These workflows for selecting statistical tests are also summarized in S3 Table. The statistical results, including *P*-values and sample numbers, are shown in the figures or corresponding legends. In the multiple comparison results, all of the *P*-values were adjusted *P*-values (Adj. *P*). S3 Table also lists the *P*-values for all the statistical tests, including those not chosen by the workflow. In this study, all the statistical tests were two-sided, and *P*-values < 0.05 was considered significant. We used statistical asterisks: *P < 0.05, **P < 0.01, ***P < 0.001, and NS for not significant.

## Supporting information

S1 Fig Increased intracellular calcium by isoflurane is derived from intracellular calcium stores. **(A, B)** Dose-dependent responses of RyR1, RyR2, and RyR3 to caffeine (**A**) and isoflurane (**B**). The peak responses (*F*_max_/*F*_0_) are shown for each (Mean ± SD). *N* = 4. Data were fitted using logistic functions (equation 4 in Materials and methods). **(C, D)** Time course reactions (**C**) and the peaks (**D**) for extracellular calcium depletion ([Ca^2+^]_E_ = 0). Each agent was given after measuring the baseline fluorescence for 10 s, as indicated by arrows. The buffer is the basal solution devoid of pharmacological agents (the vehicle control). Data are represented as Mean ± SD. *N* = 4. **(E, F)** Time course reactions (**E**) and peaks (**F**) under thapsigargin treatment. Thapsigargin was given in the nominal calcium-free medium ([Ca^2+^]_E_ = 0). Ten seconds for baseline. Data are represented as Mean ± SD. *N* = 4. TG, thapsigargin; Ionom, ionomycin.
(PDF)

S2 Fig Calcium release by isoflurane is RyR1-dependent. **(A–D)** Effects of dantrolene on caffeine and isoflurane responses in RyR1, RyR2, and RyR3. Each agent was administered after measuring the baseline fluorescence for 30 s, as indicated by arrows. The buffer is the basal solution that contains no pharmacological agents. *N* = 4. * Adj. *P* < 0.05, *** Adj. *P* < 0.001 by the Dunnett’s test or the Steel’s test. NS, not significant. **(E–H)** Effects of heterologous expression of the RyR1 brain-specific splicing variant (RyR1-BsSV) on responses to caffeine and isoflurane for RyR1, RyR2, and RyR3. *N* = 4. * Adj. *P* < 0.05, ** Adj. *P* < 0.01 by the Turkey–Kramer test or the Steel-Dwass test. NS, not significant. **(I–M)** Effects of heterologous expression of wild-type IP_3_ 5-phosphatase (5ppase(WT)) and the inactive mutant, 5ppase(R343A/R350A) on the responses of RyR1 to basal buffer (**J**), ATP (**K**), caffeine (**L**), and isoflurane (**M**). *N* = 4. *P < 0.05, ***P < 0.001 by the Student *t* test or the two-sample Wilcoxon test. Data are presented as Mean ± SD. NS, not significant.
(PDF)

S3 Fig Screening of RyR1 residues responsible for the sensitivity to isoflurane. **(A)** The isoflurane response of ChR.4 and ChR.4′. The peaks of the isoflurane response normalized by the caffeine response are shown. *N* = 4. **(B)** Isoflurane response of ChR.5–ChR.8. *N* = 4. **(C)** Isoflurane response of ChR.9–ChR.11. *N* = 4. **(D)** Isoflurane response of ChR.12 and the mutants. * Adj. *P* < 0.05, *** Adj. *P* < 0.001 by the Dunnett’s test (multiple comparisons between ChR. Twelve and other data). No stars when not significant. *N* = 8. **(E)** Isoflurane response of ChR.13 and the mutants. * Adj. *P* < 0.05, ** Adj. *P* < 0.01 by the Steel’s test (multiple comparisons between ChR. 13 and other data). No stars when not significant. *N* = 8. Data are presented as Mean ± SD. For calculating the Normalized Iso. Response (equation 1 in Materials and methods), the peaks at 5.0 mM caffeine and the control (without pharmacological agents) were normalized to 1.0 and 0, respectively.
(PDF)

S4 Fig Single amino acid substitution mutants of RyR1. **(A)** The isoflurane response of RyR1(A4004S) normalized by the caffeine response. *N* = 4. The peaks at 5.0 mM caffeine and the control (without pharmacological agents) are normalized to 1.0 and 0, respectively (equation 1 in Materials and methods). **(B)** Time-course reaction of RyR1(A4000V). Two independent constructs were tested. *N* = 4. **(C, D)** Dose–response of RyR1(WT) and RyR1(M4000F) to caffeine (**C**) and isoflurane (**D**) using tetracycline-controlled expression in their stable cell lines. The peak responses (*F*_max_/*F*_0_) are shown for each. *N* = 4. Data were fitted using logistic functions (equation 4 in Materials and methods). The RyR1(WT) data are adapted from the S1A and S1B Fig. **(E, F)** Caffeine insensitive mutant’s time course reaction (**E**) and peak intensity (**F**). *N* = 4. Data are presented as Mean ± SD.
(PDF)

S5 Fig Characterization of RyR1(M4003F) KI and RyR1-BsSV mice. **(A)** Response to caffeine and isoflurane following transient transfection of rabbit RyR1(WT), mouse RyR1(WT), and mouse RyR1(M4003F). Mean ± SD. *N* = 4. The peaks at 5.0 mM caffeine and the control (without pharmacological agents) are normalized to 1.0 and 0, respectively (equation 1 in Materials and methods). **(B)** Response to caffeine and isoflurane in cell lines expressing mouse RyR1(WT) and mouse RyR1(M4003F) under tetracycline control. Mean ± SD. *N* = 4. The data are normalized by the same procedure as in panel **A**. **(C)** Body weight of WT, heterozygous (Hetero), and homozygous (Homo) RyR1(M4003F) KI mice from siblings. *N* = 7 (WT), *N* = 10 (Hetero), and *N* = 6 (Homo). Mean ± SD with individual data points. NS, not significant (the Dunnett’s test). **(D–H)** Basal sleep phenotype for each genotype. Sibling animals were used for analysis. *N* = 6 (WT), *N* = 9 (Hetero KI), *N* = 6 (Homo KI). Hourly sleep amount (**D**), total sleep duration (**E**), *P*_ws_ (transition probability from wakefulness to sleep, equation 2 in Materials and methods) (**F**), *P*_sw_ (transition probability from sleep to wakefulness, equation 3) (**G**), and sleep episode duration (**H**) are shown, respectively. In panel **D**, the line represents the mean values, and the shaded areas are the SEM for each time point. In panels **E**–**H**, data are shown as Mean ± SD with individual data points. NS, not significant (the Dunnett’s test). **(I)** Rebound sleep following sleep deprivation (SD) of WT (*N* = 5) and Hetero KI mice (*N* = 8) from the sibling animals. Additionally to the hourly sleep amount (left), the sleep duration for 12 h after SD is indicated (right). For the hourly sleep amount, the line represents the mean values, and the shaded regions are the SEM for each time point. For the 12-h sleep duration, individual data are indicated. *P < 0.05 by paired *t* test. **(J–N)** Basal sleep phenotype of the RyR1-BsSV mice. *N* = 12 for both groups. The hourly sleep amount (**J**), total sleep duration (**K**), *P*_ws_ (**L**), *P*_sw_ (**M**), and sleep episode duration (**N**) are shown, respectively. In panel **J**, the line represents the mean values, and the shaded area is the SEM for each time point. In panels **K**–**N**, data are shown as Mean ± SD with individual data points. NS, not significant (the Welch’s t *t*est or the two-sample Wilcoxon test). ZT, zeitgeber time.
(PDF)

S6 Fig Isoflurane treatment of primary cultured cortical neurons. **(A)** Raster plots across the sequential exposure to isoflurane and the recovery in the basal culture medium and each extracellular-calcium-adjusted condition ([Ca^2+^]_E_ = 0 mM, [Ca^2+^]_E_ = 0.25 mM, [Ca^2+^]_E_ = 0.50 mM, [Ca^2+^]_E_ = 1.0 mM or [Ca^2+^]_E_ = 2.0 mM). Three independent data are indicated. **(B)** Total spike numbers normalized to the baseline (0.0% isoflurane). Mean ± SEM. *N* = 3.
(PDF)

S7 Fig Effects of phenolic compounds on RyR1. **(A)** Effects of 3-bromophenol (3-BP) and 3-bromo-5-methoxyphenol (3-B-5-M) on RyR1. *N* = 4. **(B)** Dose-dependent effects of phenol, 4-methylphenol (4-MP), and 4-ethylphenol (4-EP) on RyR1. Data were fitted using logistic functions (equation 4 in Materials and methods). *N* = 4–8. **(C)** Effects of catecholic derivatives of 4-MP (100 μM each) on RyR1. *N* = 8. **(D)** Effects of 4-methylphenyl sulfate (4-MPS) and 4-ethylphenyl sulfate (4-EPS) on RyR1. *N* = 5. Data are presented as Mean ± SD. RyR1 is expressed by tetracycline induction in the stable cell line. 4-MC, 4-methylcatechol; 4-EC, 4-ethylcatechol; 3-MC, 3-methylcatechol; 3-EC, 3-ethylcatechol.
(PDF)

S1 Table Report of the fitting variables. Fitted curves and the variables (estimates and their standard errors) are listed. H, Hill slope; and EC_50_, the half-maximal effective concentration.
(XLSX)

S2 Table Oligonucleotide sequences. The oligonucleotide sequences used in this study are listed.
(XLSX)

S3 Table Report of the statistical results. The workflow for selecting the statistical test is summarized in the initial pages. The statistical results (*P*-values) are also reported for each data set. Red shades represent the statistical results that are suggested by the workflow.
(XLSX)

S1 Data Numerical data. (XLSX)

## Abbreviations

AAV: adeno-associated virus
ATP: adenosine triphosphate
BS: burst suppression
CaMKII: Ca^2+^/calmodulin-dependent protein kinase II
ChR.: chimeric receptors
ChR.1: chimeric receptor 1
CICR: calcium-induced calcium release
DMSO: dimethyl sulfoxide
EEG: electroencephalogram
EMG: electromyogram
ER: endoplasmic reticulum
GABA_A_Rs: γ-aminobutyric acid type A receptors
HD-MEA: high-density multielectrode array
IP_3_Rs: inositol 1,4,5-trisphosphate receptors
KI: knock-in
LORR: loss of righting reflex
MACs: minimum alveolar concentrations
MH: malignant hyperthermia
MHS: malignant hyperthermia susceptibility
PDL: poly-d-lysine
PEG: polyethylene glycol
RyR1: type 1 ryanodine receptor
SD: sleep deprivation
SDW: sterile deionized water
SR: sarcoplasmic reticulum
ssODN: single-stranded oligodeoxynucleotide
SSS: Snappy Sleep Stager
TG: thapsigargin
2-BP: 2-bromophenol
3-BP: 3-bromophenol
4-BP: 4-bromophenol
4-EP: 4-ethylphenol
4-EPS: 4-ethylphenyl sulfate
4-MP: 4-methylphenol
4-MPS: 4-methylphenyl sulfate
4-PP: 4-propylphenol
