# Supplementary figures and images for: Isoflurane activates the type 1 ryanodine receptor to induce anesthesia in mice
Source: PLoS Biol. 2025 Jun 3;23(6):e3003172. doi: 10.1371/journal.pbio.3003172 (PMC12132946; doi:10.1371/journal.pbio.3003172)

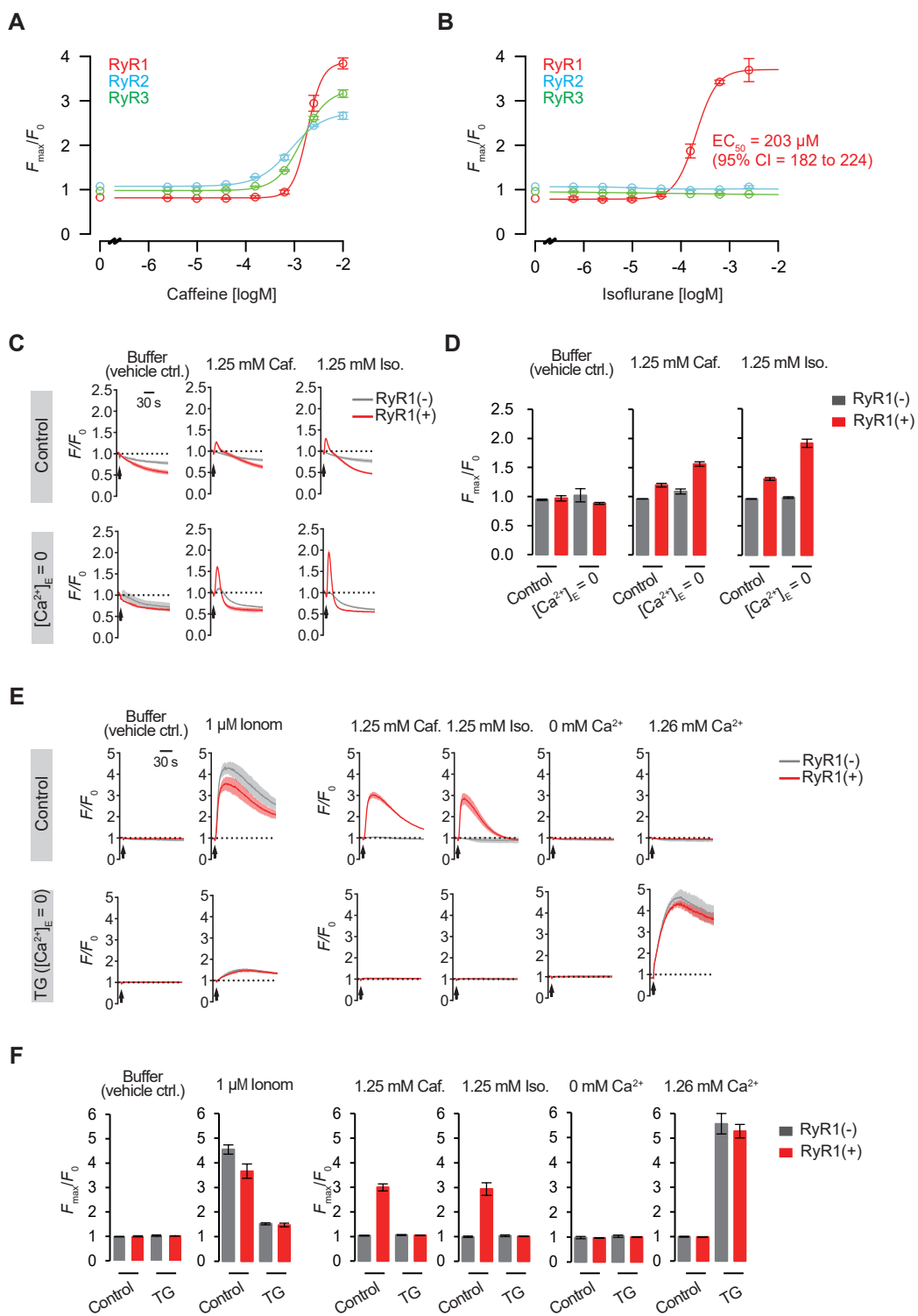

**S1 Fig. Kanaya *et al.***

Supplement: S1 Fig — (A, B) Dose-dependent responses of RyR1, RyR2, and RyR3 to caffeine (A) and isoflurane (B). The peak responses (Fmax/F0) are shown for each (Mean ± SD). N = 4. Data were fitted using logistic functions (equation 4 in Materials and methods). (C, D) Time course reactions (C) and the peaks (D) for extracellular calcium depletion ([Ca2+]E = 0). Each agent was given after measuring the baseline fluorescence for 10 s, as indicated by arrows. The buffer is the basal solution devoid of pharmacological agents (the vehicle control). Data are represented as Mean ± SD. N = 4. (E, F) Time course reactions (E) and peaks (F) under thapsigargin treatment. Thapsigargin was given in the nominal calcium-free medium ([Ca2+]E = 0). Ten seconds for baseline. Data are represented as Mean ± SD. N = 4. TG, thapsigargin; Ionom, ionomycin. (PDF) [file pbio.3003172.s001.pdf]

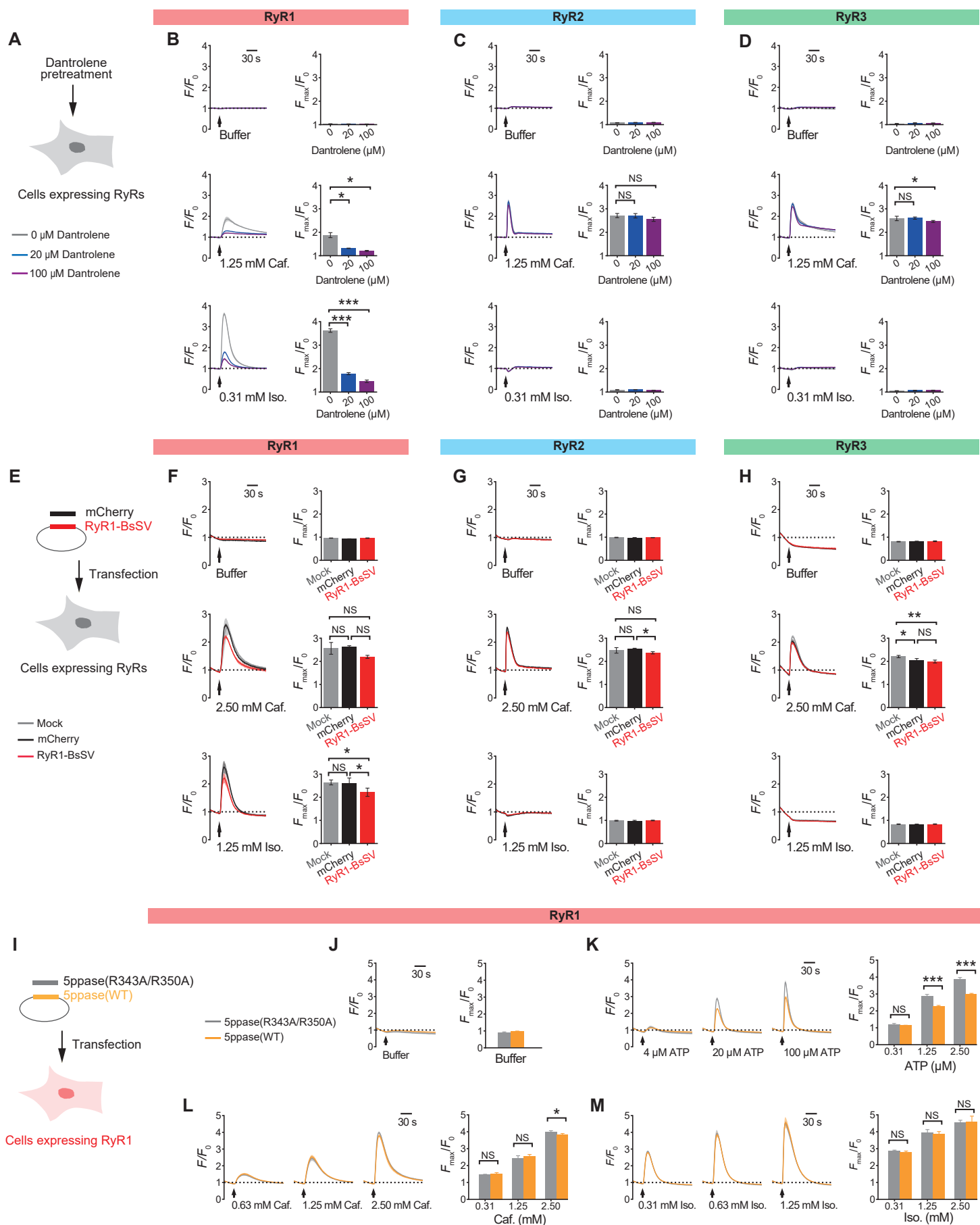

S2 Fig. Kanaya et al.

Supplement: S2 Fig — (A–D) Effects of dantrolene on caffeine and isoflurane responses in RyR1, RyR2, and RyR3. Each agent was administered after measuring the baseline fluorescence for 30 s, as indicated by arrows. The buffer is the basal solution that contains no pharmacological agents. N = 4. * Adj. P < 0.05, *** Adj. P < 0.001 by the Dunnett’s test or the Steel’s test. NS, not significant. (E–H) Effects of heterologous expression of the RyR1 brain-specific splicing variant (RyR1-BsSV) on responses to caffeine and isoflurane for RyR1, RyR2, and RyR3. N = 4. * Adj. P < 0.05, ** Adj. P < 0.01 by the Turkey–Kramer test or the Steel-Dwass test. NS, not significant. (I–M) Effects of heterologous expression of wild-type IP3 5-phosphatase (5ppase(WT)) and the inactive mutant, 5ppase(R343A/R350A) on the responses of RyR1 to basal buffer (J), ATP (K), caffeine (L), and isoflurane (M). N = 4. *P < 0.05, ***P < 0.001 by the Student t test or the two-sample Wilcoxon test. Data are presented as Mean ± SD. NS, not significant. (PDF) [file pbio.3003172.s002.pdf]

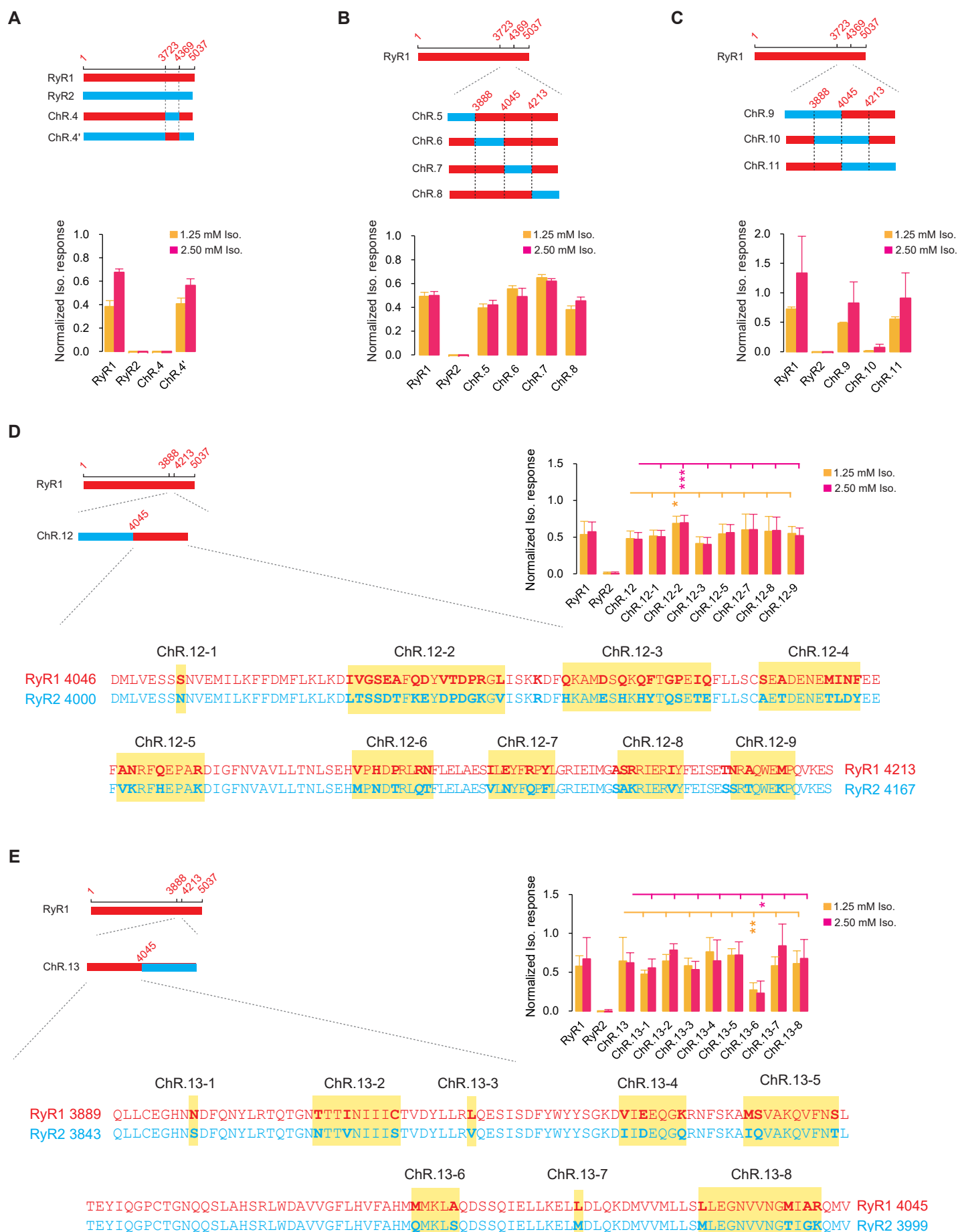

S3 Fig. Kanaya *et al.*

Supplement: S3 Fig — (A) The isoflurane response of ChR.4 and ChR.4′. The peaks of the isoflurane response normalized by the caffeine response are shown. N = 4. (B) Isoflurane response of ChR.5–ChR.8. N = 4. (C) Isoflurane response of ChR.9–ChR.11. N = 4. (D) Isoflurane response of ChR.12 and the mutants. * Adj. P < 0.05, *** Adj. P < 0.001 by the Dunnett’s test (multiple comparisons between ChR. Twelve and other data). No stars when not significant. N = 8. (E) Isoflurane response of ChR.13 and the mutants. * Adj. P < 0.05, ** Adj. P < 0.01 by the Steel’s test (multiple comparisons between ChR. 13 and other data). No stars when not significant. N = 8. Data are presented as Mean ± SD. For calculating the Normalized Iso. Response (equation 1 in Materials and methods), the peaks at 5.0 mM caffeine and the control (without pharmacological agents) were normalized to 1.0 and 0, respectively. (PDF) [file pbio.3003172.s003.pdf]

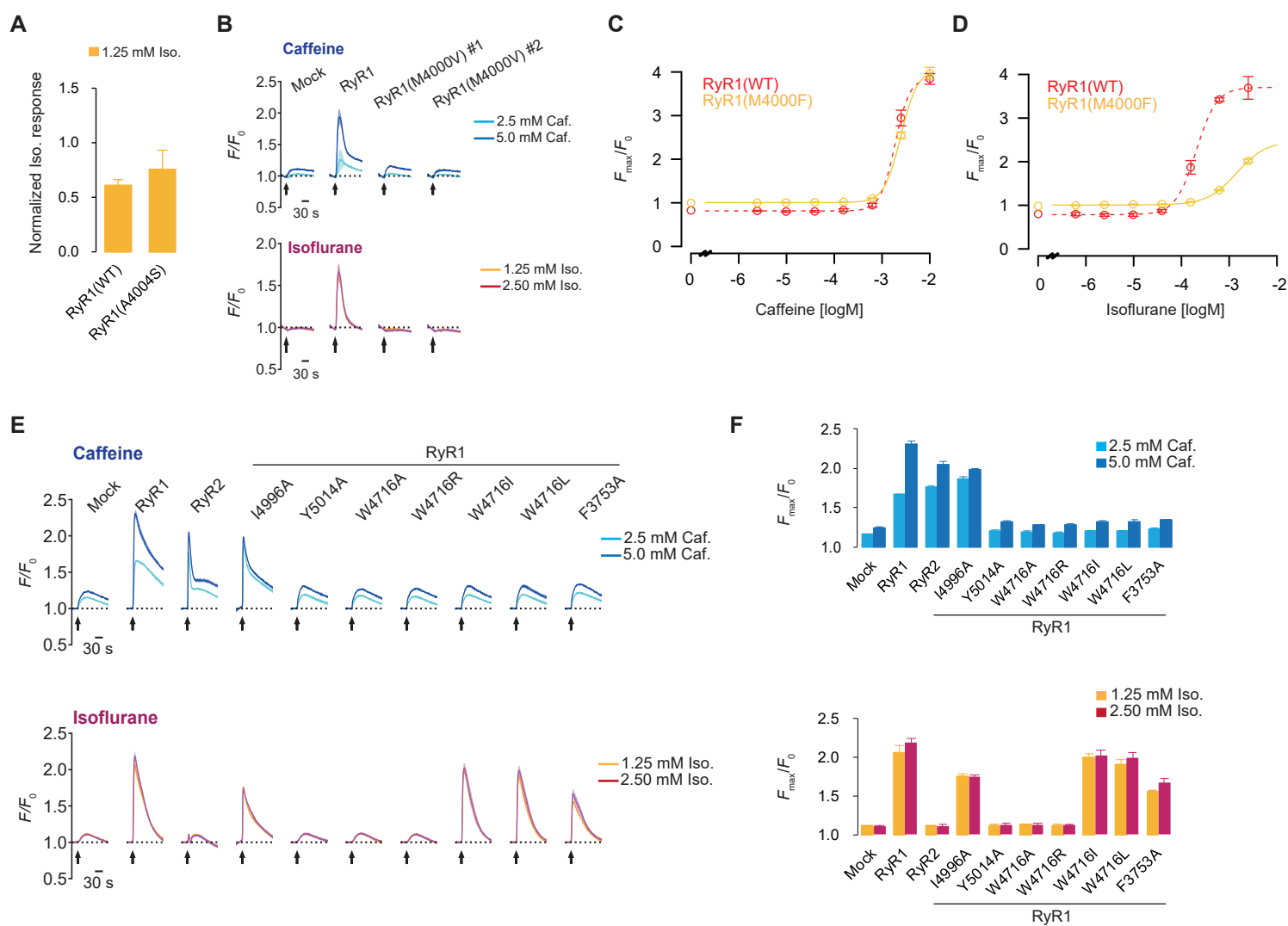

S4 Fig. Kanaya *et al.*

Supplement: S4 Fig — (A) The isoflurane response of RyR1(A4004S) normalized by the caffeine response. N = 4. The peaks at 5.0 mM caffeine and the control (without pharmacological agents) are normalized to 1.0 and 0, respectively (equation 1 in Materials and methods). (B) Time-course reaction of RyR1(A4000V). Two independent constructs were tested. N = 4. (C, D) Dose–response of RyR1(WT) and RyR1(M4000F) to caffeine (C) and isoflurane (D) using tetracycline-controlled expression in their stable cell lines. The peak responses (Fmax/F0) are shown for each. N = 4. Data were fitted using logistic functions (equation 4 in Materials and methods). The RyR1(WT) data are adapted from the S1A and S1B Fig. (E, F) Caffeine insensitive mutant’s time course reaction (E) and peak intensity (F). N = 4. Data are presented as Mean ± SD. (PDF) [file pbio.3003172.s004.pdf]

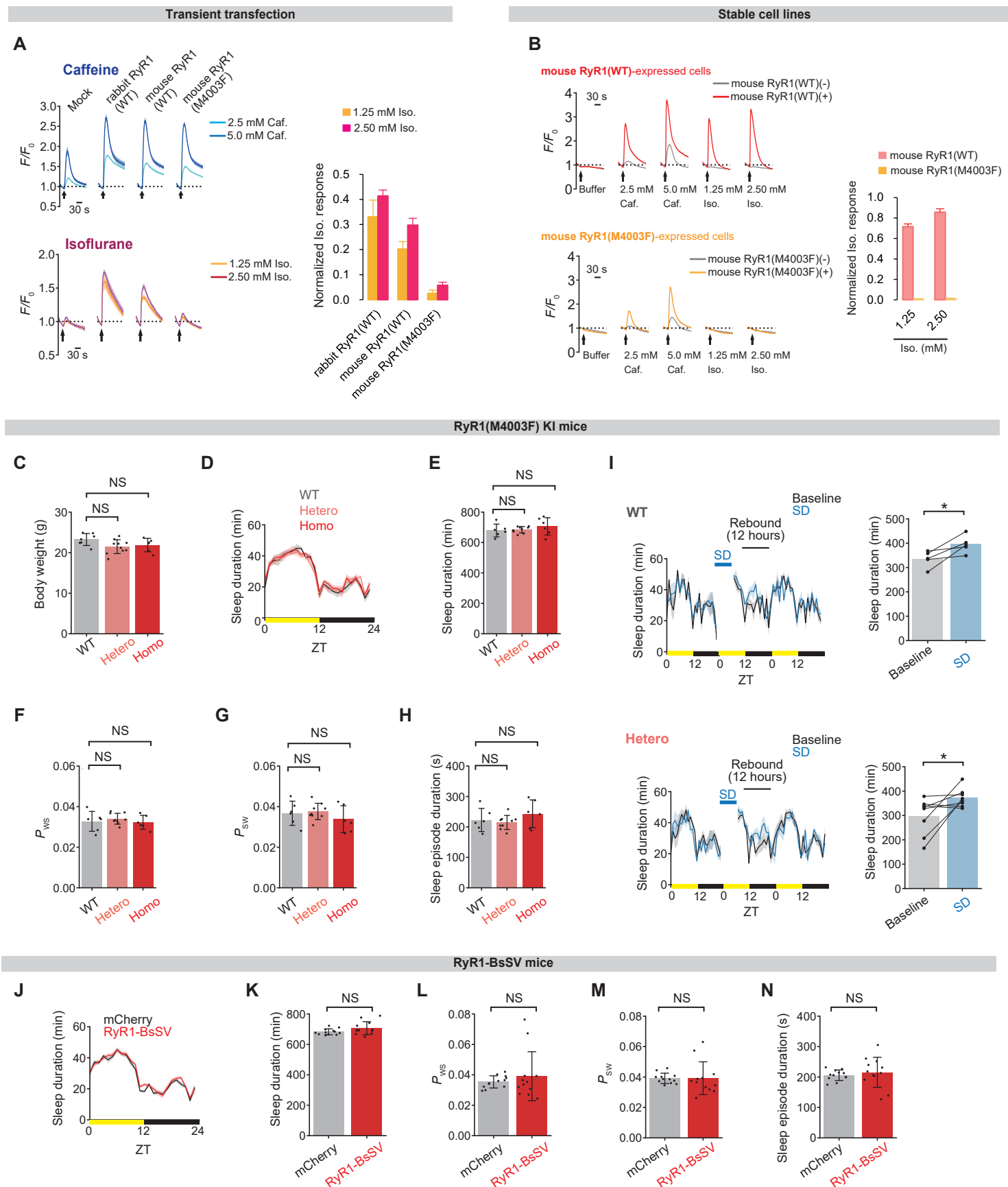

**S5 Fig. Kanaya et al.**

Supplement: S5 Fig — (A) Response to caffeine and isoflurane following transient transfection of rabbit RyR1(WT), mouse RyR1(WT), and mouse RyR1(M4003F). Mean ± SD. N = 4. The peaks at 5.0 mM caffeine and the control (without pharmacological agents) are normalized to 1.0 and 0, respectively (equation 1 in Materials and methods). (B) Response to caffeine and isoflurane in cell lines expressing mouse RyR1(WT) and mouse RyR1(M4003F) under tetracycline control. Mean ± SD. N = 4. The data are normalized by the same procedure as in panel A. (C) Body weight of WT, heterozygous (Hetero), and homozygous (Homo) RyR1(M4003F) KI mice from siblings. N = 7 (WT), N = 10 (Hetero), and N = 6 (Homo). Mean ± SD with individual data points. NS, not significant (the Dunnett’s test). (D–H) Basal sleep phenotype for each genotype. Sibling animals were used for analysis. N = 6 (WT), N = 9 (Hetero KI), N = 6 (Homo KI). Hourly sleep amount (D), total sleep duration (E), Pws (transition probability from wakefulness to sleep, equation 2 in Materials and methods) (F), Psw (transition probability from sleep to wakefulness, equation 3) (G), and sleep episode duration (H) are shown, respectively. In panel D, the line represents the mean values, and the shaded areas are the SEM for each time point. In panels E–H, data are shown as Mean ± SD with individual data points. NS, not significant (the Dunnett’s test). (I) Rebound sleep following sleep deprivation (SD) of WT (N = 5) and Hetero KI mice (N = 8) from the sibling animals. Additionally to the hourly sleep amount (left), the sleep duration for 12 h after SD is indicated (right). For the hourly sleep amount, the line represents the mean values, and the shaded regions are the SEM for each time point. For the 12-h sleep duration, individual data are indicated. *P < 0.05 by paired t test. (J–N) Basal sleep phenotype of the RyR1-BsSV mice. N = 12 for both groups. The hourly sleep amount (J), total sleep duration (K), Pws (L), Psw (M), and sleep episode duration (N) are sh [file pbio.3003172.s005.pdf]

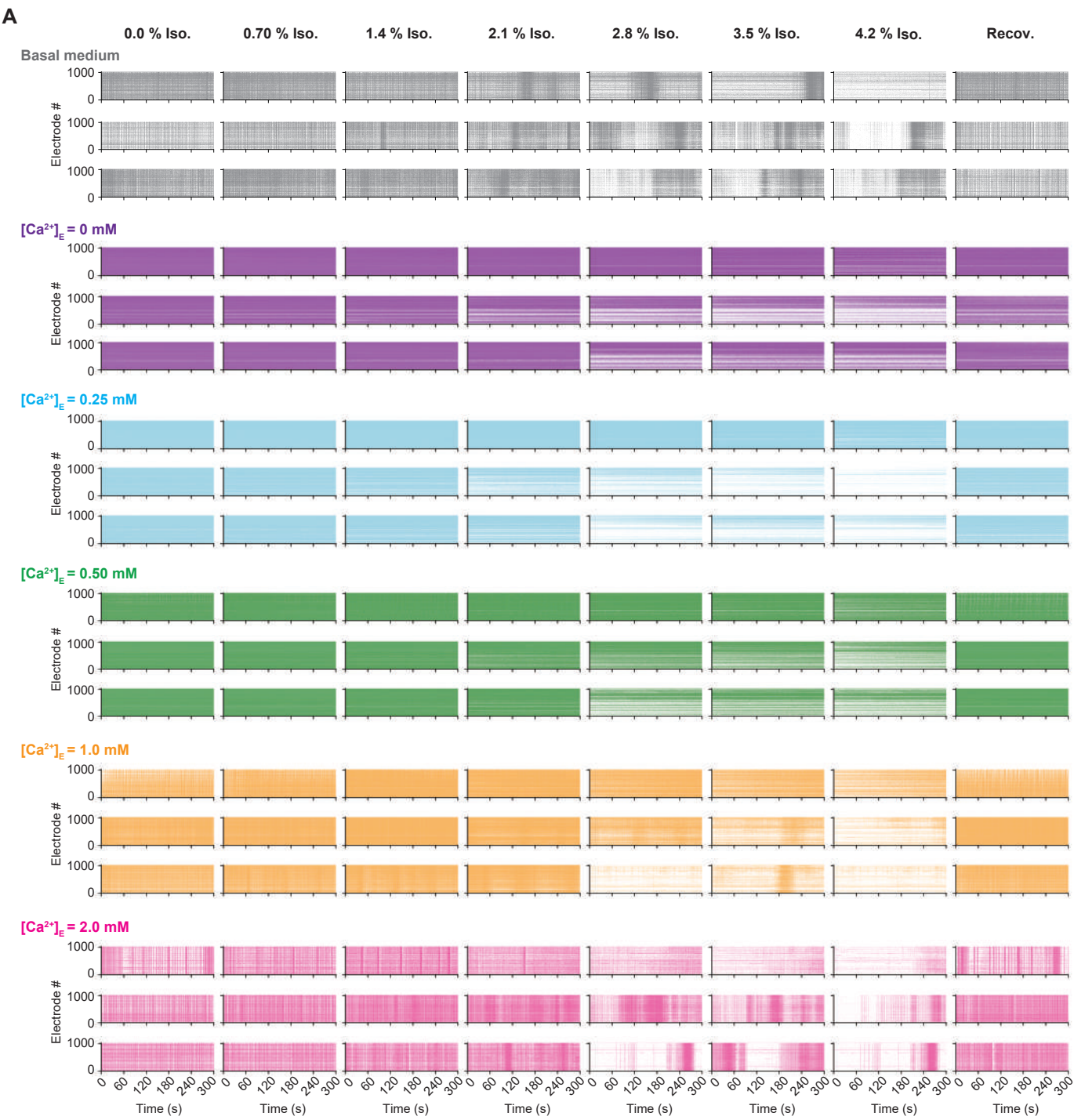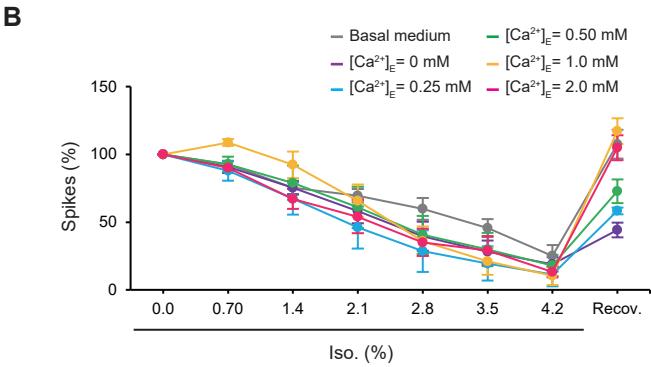

S6 Fig. Kanaya *et al.*

Supplement: S6 Fig — (A) Raster plots across the sequential exposure to isoflurane and the recovery in the basal culture medium and each extracellular-calcium-adjusted condition ([Ca2+]E = 0 mM, [Ca2+]E = 0.25 mM, [Ca2+]E = 0.50 mM, [Ca2+]E = 1.0 mM or [Ca2+]E = 2.0 mM). Three independent data are indicated. (B) Total spike numbers normalized to the baseline (0.0% isoflurane). Mean ± SEM. N = 3. (PDF) [file pbio.3003172.s006.pdf]

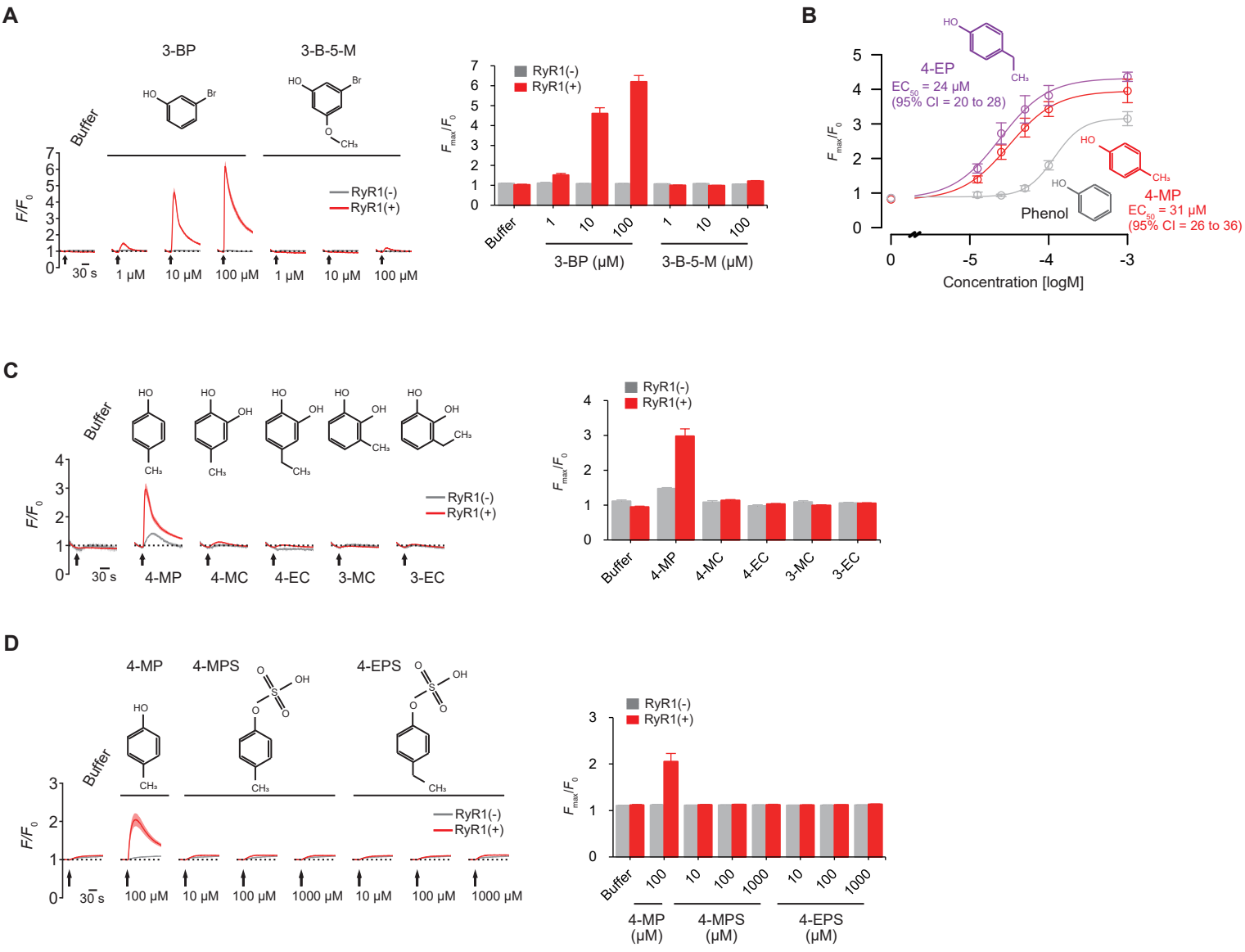

S7 Fig. Kanaya *et al.*

Supplement: S7 Fig — (A) Effects of 3-bromophenol (3-BP) and 3-bromo-5-methoxyphenol (3-B-5-M) on RyR1. N = 4. (B) Dose-dependent effects of phenol, 4-methylphenol (4-MP), and 4-ethylphenol (4-EP) on RyR1. Data were fitted using logistic functions (equation 4 in Materials and methods). N = 4–8. (C) Effects of catecholic derivatives of 4-MP (100 μM each) on RyR1. N = 8. (D) Effects of 4-methylphenyl sulfate (4-MPS) and 4-ethylphenyl sulfate (4-EPS) on RyR1. N = 5. Data are presented as Mean ± SD. RyR1 is expressed by tetracycline induction in the stable cell line. 4-MC, 4-methylcatechol; 4-EC, 4-ethylcatechol; 3-MC, 3-methylcatechol; 3-EC, 3-ethylcatechol. (PDF) [file pbio.3003172.s007.pdf]
